# Impact of pesticide regulations on mortality from suicide by pesticide in China: an interrupted time series analysis

**DOI:** 10.3389/fpsyt.2023.1189923

**Published:** 2023-09-04

**Authors:** Yongfu Yan, Yingying Jiang, Rong Liu, Michael Eddleston, Chuanjiang Tao, Andrew Page, Lijun Wang, Guoshuang Feng, Shiwei Liu

**Affiliations:** ^1^Tobacco Control Office, Chinese Center for Disease Control and Prevention, Beijing, China; ^2^National Center for Chronic and Noncommunicable Disease Control and Prevention, Chinese Center for Disease Control and Prevention, Beijing, China; ^3^The George Institute for Global Health, Faculty of Medicine, The University of New South Wales (UNSW), Sydney, NSW, Australia; ^4^Centre for Pesticide Suicide Prevention, University of Edinburgh, Edinburgh, United Kingdom; ^5^Institute for the Control of Agrochemicals, Ministry of Agriculture and Rural Affairs, Beijing, China; ^6^Translational Health Research Institute, Western Sydney University, Penrith, NSW, Australia; ^7^Big Data Center, Beijing Children's Hospital, Capital Medical University, National Center for Children's Health, Beijing, China

**Keywords:** epidemiology, impact evaluation, pesticide, suicide, interrupted time series design

## Abstract

**Background:**

Pesticide bans and regulatory restrictions have been shown to be effective strategies for preventing suicide in several countries. Suicide and suicide by pesticides have decreased significantly in China over the past two decades. However, whether the reduction was associated with pesticide regulation is unknown.

**Methods:**

The monthly data on suicide and suicide by pesticide from 2006 to 2018 were obtained from China's Disease Surveillance Point (DSP) system. Information on China's pesticide regulations since 1970 was obtained from Pesticide Action Network International (PAN International), Joint Meeting on Pesticide Management Highly Hazardous Pesticides (JMPM HHP) lists, the website of the Ministry of Agriculture of China, Pesticide Information Network of China, and the Wan Fang database. Change point detection and policy analysis were combined to identify the time of any trend change breakpoint of suicide and suicide by pesticide. Interrupted time series analysis was used to investigate the pre- and post-breakpoint trends of monthly standardized rates in suicide and suicide by pesticide.

**Results:**

The standardized pesticide suicide rate decreased by 60.5% from 6.50 in 2006 to 2.56 per 100,000 in 2018. Larger declines were evident among people in urban areas (67.3%), female individuals (63.5%), and people aged 15–44 years (68.1%). The effect of policies banning highly hazardous organophosphorus pesticides (HHOP) [rate ratio (RR) = 0.993, 95% CIs (0.991–0.994)] in December 2008 and stopping domestic sales and use of paraquat aqueous solution (RR = 0.992, 95% CIs: 0.990–0.994) in July 2016 were more pronounced than regulating the paraquat-related products (RR = 1.003, 95% CIs: 1.002–1.004) in April 2012.

**Conclusion:**

Declines in suicide by pesticide in China occurred contemporaneously with regulatory bans and restrictions implemented on several pesticides, particularly in urban areas, among female individuals, and the relatively low age profile. These findings indicate the potential influence of these bans on trends of suicide by pesticides.

## 1. Introduction

Suicide is a serious and important global health problem. There were approximately 703,000 suicides worldwide in 2019 (an age-standardized rate of 9 per 100,000 people), accounting for 1.3% of all deaths and ranking as the 17th leading cause of death globally (1, 2). Importantly, 79% of these suicides occurred in low- and middle-income countries (3). To effectively address this issue, multi-sectoral organizations and relevant stakeholders have been encouraged to take concerted action through comprehensive national strategies and measures (4–6). The World Health Organization's (WHO) Comprehensive Mental Health Action Plan 2013–2030 and Sustainable Development Goal 3 (target 3.4 and indicator 3.4.2) set out the global goal of reducing suicide rates by one-third by 2030 (3, 7).

Despite declines in the rate of suicide in the past two decades, the number of suicides in China is still very large compared to the rest of the world (3, 8–12). Research suggests that a substantial proportion of suicidal acts in China are impulsive, following acute psychosocial stress or crisis (13). The leading method of suicide in China is pesticide poisoning, accounting for approximately half of all suicide deaths (9, 14). It is widely recognized that one of the most effective approaches for preventing suicide is restricting the availability of commonly used, high-lethality methods (15). In countries where pesticides account for a high proportion of suicides, such as China, the WHO, and the Food and Agriculture Organization of the United Nations (FAO) currently recommend the pesticide regulator identify, and withdraw from the sale, the pesticides most commonly used in fatal self-poisoning (16). These recommendations are supported by a systematic review of the impact of pesticide bans on suicides by pesticide poisoning as well as overall suicide rates, which is best exemplified by evaluations of bans in South Korea and Sri Lanka, which have both enacted bans on highly hazardous pesticides in recent years (17–19). Cost-effectiveness analysis shows that national bans of highly hazardous pesticides are highly cost-effective for reducing suicide by pesticides (20).

In China, pesticide regulation began in the 1970s (Table 1) (21). Local evaluations on the association between pesticide exposure and suicide attempt patterns have been undertaken in several provinces in China (22–25). The impact of these bans has not been investigated at a national level. This study (1) collates information on the array of pesticide bans and regulatory changes since 1970 and (2) investigates the association between pesticide bans and regulations on national trends of suicide in China.

**Table 1:** Banned or restricted pesticides and issued years in China.

**Pesticides**	**Regulations**	**Policy measure and strength**	**Implementation year**
Arsenic compounds	NA	NA	1970
Fluoroacetamide	Regulations on the safe use of pesticides issued jointly by the former Ministry of Agriculture, Animal Husbandry and Fisheries and the Ministry of Health	***Warning*****:** the regulations provide the classification, use, purchase, transportation, storage, precautions, application of personnel selection, and personal protection of pesticides, in which fluoroacetamide is classified as one of the riskiest pesticides that can cause poisoning or death even on exposure to a minimal amount.	1982
Ethylene dibromide/EDB/1,2-dibromoethane	NA	NA	1984
Aldicarb	Announcement No. 194 of the Ministry of Agriculture of the People's Republic of China	***Stops*****:** registrations and new applications of aldicarb; ***Revoking*****:** the registration of aldicarb in the apple tree.	2002
Carbofuran	Announcement No. 194 of the Ministry of Agriculture of the People's Republic of China	***Stops*****:** registrations and new applications of carbofuran; ***Revoking*****:** the registration of carbofuran in the tangerine tree.	2002
Coumaphos	Announcement No. 199 of the Ministry of Agriculture of the People's Republic of China	***Ban*****:** being used for vegetables, fruits, tea, and herbal medicine materials.	2002
DDT	Announcement No. 199 of the Ministry of Agriculture of the People's Republic of China	***Prohibits*****:** use.	2002
Ethoprophos/Ethoprop	Announcement No. 199 of the Ministry of Agriculture of the People's Republic of China	***Ban*****:** being used for vegetables, fruits, tea, and herbal medicine materials.	2002
Fenamiphos	Announcement No. 199 of the Ministry of Agriculture of the People's Republic of China	***Ban*****:** being used for vegetables, fruits, tea, and herbal medicine materials.	2002
Fonophos/fonofos	Announcement No. 199 of the Ministry of Agriculture of the People's Republic of China	***Ban*****:** being used for vegetables, fruits, tea, and herbal medicine materials.	2002
Gliftor	Announcement No. 199 of the Ministry of Agriculture of the People's Republic of China	***Prohibits*****:** use.	2002
Hexachlorocyclohexane (HCH)	Announcement No. 199 of the Ministry of Agriculture of the People's Republic of China	***Prohibits*****:** use.	2002
Isocarbophos	Announcement No. 194 of the Ministry of Agriculture of the People's Republic of China	***Stops*****:** registrations and new applications of Isocarbophos.	2002
Isofenphos-methyl/Isophenphos-methyl	Announcement No. 199 of the Ministry of Agriculture of the People's Republic of China	***Ban*****:** being used for vegetables, fruits, tea, and herbal medicine materials.	2002
Mercury compounds	Announcement No. 199 of the Ministry of Agriculture of the People's Republic of China	***Prohibits*****:** use.	2002
Methomyl	Announcement No. 194 of the Ministry of Agriculture of the People's Republic of China	***Stops*****:** registrations and new applications of methomyl.	2002
Omethoate	Announcement No. 194 of the Ministry of Agriculture of the People's Republic of China	***Stops*****:** registrations and new applications of Omethoate.	2002
Phorate	Announcement No. 194 of the Ministry of Agriculture of the People's Republic of China	***Stops*****:** registrations and new applications of phorate.	2002
Phosfolan-methyl	Announcement No. 194 of the Ministry of Agriculture of the People's Republic of China	***Stops*****:** registrations and new applications of Phosfolan-methyl.	2002
Silatrane	Announcement No. 199 of the Ministry of Agriculture of the People's Republic of China	***Prohibits*****:** use.	2002
Sodium fluoroacetate/1080	Announcement No. 199 of the Ministry of Agriculture of the People's Republic of China	***Prohibits*****:** use.	2002
Sulfotep	Announcement No. 194 of the Ministry of Agriculture of the People's Republic of China	***Stops*****:** registrations and new applications of Sulfotep.	2002
Terbufos	Announcement No. 194 of the Ministry of Agriculture of the People's Republic of China	***Stops*****:** registrations and new applications of terbufos.	2002
Tetramine	Announcement No. 199 of the Ministry of Agriculture of the People's Republic of China	***Prohibits*****:** use.	2002
Daminozide	Announcement No. 274 of the Ministry of Agriculture of the People's Republic of China	***Revoking*****:** the registration of daminozide on peanut; ***Prohibits*****:** daminozide pesticide products on peanut.	2003
Methamidophos	Announcement No. 274 of the Ministry of Agriculture of the People's Republic of China	***Stops*****:** registrations of mixed preparations containing methamidophos and renewal registration of temporary registration of the single dose of expiration of 4 years (2003) and sale (2004); ***Revoking*****:** the registration of mixed preparations containing methamidophos; ***Prohibits*****:** sale mixed preparations containing methamidophos on the market.	2003
Methyl parathion	Announcement No. 274 of the Ministry of Agriculture of the People's Republic of China	***Stops*****:** registrations of mixed preparations containing methyl parathion and renewal registration of temporary registration of the single dose of expiration of 4 years (2003) and sale (2004); ***Revoking*****:** the registration of mixed preparations containing methyl parathion; ***Prohibits*****:** sale mixed preparations containing Methyl parathion on the market.	2003
Monocrotophos	Announcement No. 274 of the Ministry of Agriculture of the People's Republic of China	***Stops*****:** registrations of mixed preparations containing monocrotophos and renewal registration of temporary registration of the single dose of expiration of 4 years (2003) and sale (2004); ***Revoking*****:** the registration of mixed preparations containing monocrotophos; ***Prohibits*****:** sale mixed preparations containing Monocrotophos on the market.	2003
Parathion (ethyl)	Announcement No. 274 of the Ministry of Agriculture of the People's Republic of China	***Stops*****:** registrations of mixed preparations containing parathion (ethyl) and renewal registration of temporary registration of the single dose of expiration of 4 years (2003) and sale (2004); ***Revoking*****:** the registration of mixed preparations containing parathion (ethyl); ***Prohibits*****:** sale mixed preparations containing Parathion (ethyl) on the market.	2003
Phosphamidon	Announcement No. 274 of the Ministry of Agriculture of the People's Republic of China	***Stops*****:** registrations of mixed preparations containing Phosphamidon and renewal registration of temporary registration of the single dose of expiration of 4 years (2003) and sale (2004); ***Revoking*****:** the registration of mixed preparations containing Phosphamidon; ***Prohibits*****:** sale mixed preparations containing Phosphamidon on the market.	2003
Pentachlorophenol (PCP) and salts	Stockholm Convention on Persistent Organic Pollutants		2004
Chlorsulfuron	Announcement No. 671 of the Ministry of Agriculture of the People's Republic of China	***Stops*****:** registrations and new applications of herbicide products containing chlorsulfuron (including the original drug, single dose, and combination preparations); ***Limit*****:** for those products containing chlorsulfuron approved on wheat, the dosage of effective components of chlorsulfuron shall not exceed 15G/HA(1G/mu).	2006
Ethametsulfuron	Announcement No. 671 of the Ministry of Agriculture of the People's Republic of China	***Stops*****:** registrations and new applications of herbicide products containing ethametsulfuron (including the original drug, single dose, and combination preparations).	2006
Metsulfuron-methyl	Announcement No. 671 of the Ministry of Agriculture of the People's Republic of China	***Stops*****:** registrations and new applications of herbicide products containing metsulfuron-methyl (including the original drug, single dose, and combination preparations); ***Limit*****:** for those products containing metsulfuron-methyl approved on wheat, the dosage of effective components of chlorsulfuron shall not exceed 7.5G/HA (0.5G/mu).	2006
Methamidophos, parathion-methyl, parathion, monocrotophos, ammonium phosphate	Joint notice No.1 by National Development and Reform Commission, State Administration for Industry and Commerce, General Administration of Quality Supervision Inspection and Quarantine	***Revoking*****:** the registrations, production licenses, and production approval certificates of methamidophos, parathion-methyl, parathion, monocrotophos, and ammonium phosphate; ***Prohibits*****:** their domestic production, circulation, and using on their own or mixed with other substances.	2008
Fipronil	Announcement No. 1157 of the Ministry of Agriculture of the People's Republic of China	***Stops*****:** processing and approving the field trial; ***Revoking*****:** registration (including formal registration, repacking registration and provisional registration of pesticide formulations containing fipronil ingredient, and repeals their registration and production approval certificate and domestic sales, except for health, for corn seed coating agent, and for the export products.)	2009
Hexachlorobenzene/Benzene hexachloride (HCB/BHC)	Ministry of Environmental Protection Announcement No. 23	***Prohibits*****:** domestic production, circulation, use, import, and export of hexachlorobenzene/benzene hexachloride (HCB/BHC), except for emergencies.	2009
Aluminum phosphide	Joint Announcement No. 1586 of the Ministry of Agriculture, the Ministry of Industry and Information Technology, the Ministry of Environmental Protection, the state administration for industry and commerce, and the State Administration for Quality Supervision, Inspection and Quarantine	***Stops*****:** new applications for field trials, applications for registration and production license, and the approval of new registration and pesticide production license (production approval document) of aluminum phosphide.	2011
Cadusafos	Joint Announcement No. 1586 of the Ministry of Agriculture, the Ministry of Industry and Information Technology, the Ministry of Environmental Protection, the state administration for industry and commerce and the State Administration for Quality Supervision, Inspection and Quarantine	***Stops*****:** new applications for field trials, applications for registration and production license, and the approval of new registration and pesticide production license (production approval document) of cadusafos.	2011
Calcium phosphide	Joint Announcement No. 1586 of the Ministry of Agriculture, the Ministry of Industry and Information Technology, the Ministry of Environmental Protection, the state administration for industry and commerce and the State Administration for Quality Supervision, Inspection and Quarantine	***Stops*****:** new applications for field trials, applications for registration and production license, and the approval of new registration and pesticide production license (production approval document) of calcium phosphide.	2011
Endosulfan	Joint Announcement No. 1586 of the Ministry of Agriculture, the Ministry of Industry and Information Technology, the Ministry of Environmental Protection, the state administration for industry and commerce and the State Administration for Quality Supervision, Inspection and Quarantine	***Stops*****:** new applications for field trials, applications for registration and production license, and the approval of new registration and pesticide production license (production approval document) of endosulfan (2011) and sale and use (2013).	2011
Magnesium phosphide	Joint Announcement No. 1586 of the Ministry of Agriculture, the Ministry of Industry and Information Technology, the Ministry of Environmental Protection, the state administration for industry and commerce and the State Administration for Quality Supervision, Inspection and Quarantine	***Stop*****:** new applications for field trials, applications for registration and production license, and the approval of new registration and pesticide production license (production approval document) of magnesium phosphide (2011) and sale and use (2013).	2011
Methidathion	Joint Announcement No. 1586 of the Ministry of Agriculture, the Ministry of Industry and Information Technology, the Ministry of Environmental Protection, the state administration for industry and commerce and the State Administration for Quality Supervision, Inspection and Quarantine	***Stops*****:** new applications for field trials, applications for registration and production license, and the approval of new registration and pesticide production license (production approval document) of methidathion (2011) and sale and use (2013).	2011
Methyl bromide	Joint Announcement No. 1586 of the Ministry of Agriculture, the Ministry of Industry and Information Technology, the Ministry of Environmental Protection, the state administration for industry and commerce and the State Administration for Quality Supervision, Inspection and Quarantine	***Stops*****:** new applications for field trials, applications for registration and production license, and the approval of new registration and pesticide production license (production approval document) of methyl bromide (2011) and sale and use (2013).	2011
Zinc phosphide	Joint Announcement No. 1586 of the Ministry of Agriculture, the Ministry of Industry and Information Technology, the Ministry of Environmental Protection, the state administration for industry and commerce and the State Administration for Quality Supervision, Inspection and Quarantine	***Stops*****:** new applications for field trials, applications for registration and production license, and the approval of new registration and pesticide production license (production approval document) of zinc phosphide (2011) and sale and use (2013).	2011
Paraquat	Ministry of Agriculture, Ministry of Industry and Information Technology and General Administration of Quality Supervision, Inspection and Quarantine Announcement No. 1745	***Stops*****:** new paraquat parent drug and aqueous solution registration and production (2012), domestic sales and use of paraquat aqueous solution (2016); ***Revoking*****:** registration and license of paraquat aqueous solution (2014); ***Adding:*** sufficient emetic, odorizer, and colorant in paraquat. Manufacturer after-sales service of application guide and poison rescuing.	2012
Asomate	Announcement No. 2032 of the Ministry of Agriculture of the People's Republic of China	***Stops*****:** the application for registration and new pesticide registration of asomate; ***Revoking*****:** asomate's registration; ***Prohibits*****:** domestic sale and use (2015).	2014
Chlorpyrifos	Announcement No. 2032 of the Ministry of Agriculture of the People's Republic of China	***Stops*****:** the application for registration on vegetables and new pesticide registration of chlorpyrifos (2016); ***Revoking*****:** chlorpyrifos' registration on vegetables and forbids its use on vegetables.	2014
Triazophos	Announcement No. 2032 of the Ministry of Agriculture of the People's Republic of China	***Stops*****:** the application for registration on vegetables and new pesticide registration of triazophos; ***Revoking*****:** triazophos' registration on vegetables (2014) and forbids its use on vegetables (2016).	2014
Urbacide	Announcement No. 2032 of the Ministry of Agriculture of the People's Republic of China	***Stops*****:** the application for registration and new pesticide registration of urbacide; ***Revoking*****:** urbacide's registration and forbids its domestic sale and use. ***Prohibits*****:** domestic sale and use (2015).	2014
Chloropicrin	Announcement No. 2289 of the Ministry of Agriculture of the People's Republic of China	***Change*****:** the use scope and application methods of registration of chloropicrin to soil fumigation; ***Revoking*****:** other registration except for soil fumigation.	2015
Acephate	Announcement No. 2552 of the Ministry of Agriculture of the People's Republic of China	***Stops*****:** processing and approving its registration on vegetables, fruits, tea, mushrooms, and herbs materials; ***Revoking*****:** the registration on vegetables, fruits, tea, mushrooms and herbs materials of acephate; ***Prohibits*****:** use of vegetables, fruits, tea, mushrooms and herbs materials.	2017
Sulfluramid	Announcement No. 148 of the Ministry of Agriculture and Rural Affairs of the People's Republic of China	***Stops*****:** processing, approving registration, and registering extension pesticide products containing sulfluramid; ***Revoking*****:** sulfluramid registration, production permit; ***Prohibits*****:** domestic use (2020).	2019
Paraquat	Notice of the Ministry of Agriculture and Rural Affairs of the People's Republic of China	***Prohibits*****:** use of paraquat sol.	2020

NA, not available. The times in parentheses indicate the execution time of the partial policy content.

## 2. Materials and methods

### 2.1. Suicide and population data

Suicide data were obtained from the China National Disease Surveillance Point (DSP) system for the period 2006–2018. The DSP system of cause-of-death data developed into a national and regionally representative sample vital registration system in 1990 with 145 DSPs. In 2004, the number of DSPs increased from 145 to 161, and the cause-of-death data were collected through a household survey before being reported to the local CDC. In 2013, the DSP system further expanded and the number of DSPs increased to 605, covering approximately 24% of the Chinese people with provincial representativeness (26). Cases of death in the DSP system are collected from hospitals and from household surveys to capture non-hospital deaths. All the data obtained are exchanged and certified with relevant departments, including civil affairs, public security, and the maternal and child healthcare sector (26, 27). In this study, 158 DSPs that are consistent in the surveillance system from January 2006 to December 2018 were included in the analysis (with 3 DSPs excluded since 2013). Population data were derived from the National Statistical Bureau of China, and suicide rates were age-standardized using the standard population of China's 2010 census. Suicide counts in this study were defined based on the International Classification of Diseases 10th Revision (ICD-10) codes X60-X84, Y10-Y34, W75-W76, and X48. ICD-10 codes for counts of suicide by pesticide were X68 (intentional pesticide suicide), X48 (accidental pesticide suicide), and Y18 (intentional unknown pesticide suicide). Deaths by accidental pesticide (X48) poisoning were also included, as research indicates many of these deaths may be missed suicides (28).

### 2.2. Pesticide regulation data

Data on China's pesticide bans and regulations were collated using several sources. The key source was data from the Pesticide Action Network International (PAN International) (29–31). Information was also obtained from the FAO/WHO Joint Meeting on Pesticide Management Highly Hazardous Pesticide (JMPM HHP) lists (31). The website of the Ministry of Agriculture of China, the China Pesticide Information Network, and the Wan Fang database were used as additional sources as well as the personal notes of one of the authors (CJT). The search period for pesticide bans and regulations related to the earliest available period (1970) to 31 December 2021.

### 2.3. Definition of the intervention point

The intervention points were defined by combined change point detection (CPD) and content analysis of the pesticide policy. Altogether, 54 pesticides have been banned and restricted in China since 1970, with almost half [*n* = 24 (44.4%)] of them being banned or restricted in 2002 (Table 1). However, for many of the key highly hazardous organophosphorus pesticides (HHOP) used for self-harm, the years 2002–2004 represented only restrictions on registration. The pesticides were available (although at likely lower levels) for agriculture until their ultimate ban in December 2008. Therefore, December 2008 was defined as the primary intervention point for subsequent time-series models. The CPD method estimates the model by fitting iteratively the linear model and was implemented by the *segmented* package in R (32, 33) and explored the monthly location of suicide by pesticides breakpoints, assuming that 1–4 breakpoints existed in 2006–2018 (Appendix Table 1). Considering the content of the policy and its implementation, we identified the potentially effective policies, including banning HHOP in December 2008, stopping registration and production of new paraquat parent drug and aqueous solutions, regulating the existing paraquat production and after-sales services in April 2012, and stopping domestic sales and use of paraquat aqueous solution in July 2016. We analyzed the three breakpoints corresponding to the abovementioned policies (Table 1).

### 2.4. Statistical analysis

An interrupted time series (ITS) regression method was used in this study to evaluate the impact of the enactment of pesticide regulations on suicide in China (34–36). Our null hypothesis was that the intervention point was not associated with any changes in suicides by pesticide among adults aged 15 years and older in China. Segmented regression analysis was conducted for the analysis of three intervention policies (37). Generalized least squares method and fitting linear segmented model (function *gls* in R package *nlme*) were used to investigate the relationship between the standardized monthly number of deaths, interventions, and time (38). We considered January 2006 as the starting time, December 2008 as intervention 1, April 2012 as intervention 2, and July 2016 as intervention 3 to investigate whether trends of suicide changed after the pesticide regulations. The final regression equation is as follows:


$$\begin{array}{l} log\left(S_{t}\right)=\beta_{0}+offset\left(log\left(people_{t}\right)\right)+\beta_{1}time+\beta_{2}intervention_{1}+\\ \;\beta_{3}(time-T_{1})\;*\;intervention_{1}+\beta_{4}intervention_{2}+\\ \;\beta_{5}(time-T_{2})\;*\;intervention_{2}+\beta_{6}intervention_{3}+\\ \;\beta_{7}(time-T_{3})\;*\;intervention_{3}+\beta_{8-13}fourier+\;\varepsilon_{t}, \end{array}$$

where *S*_*t*_ is the standardized monthly suicide number in time *t*, *people*_*t*_ is the number of standard population (in person-years) with log-transformed and was used as an offset term in time *t*, β_0_ is the intercept, *time* is the corresponding month and year calculated in cumulative months, and β_1_ represents the underlying pre-intervention trend. *intervention*_1_, *intervention*_2_, and *intervention*_3_ were coded as a binary variable to reflect the pesticide policy enactment (pre-intervention period: 0; began and post-intervention period: 1), and the corresponding regression coefficients β_2_, β_4_, and β_6_ indicate the level change and reflect the immediate effect following interventions 1–3, respectively. *T*_1_, *T*_2_, and *T*_3_ were the time points when interventions started. (*time*−*T*_1_)*intervention_1_, (*time*−*T*_2_)*intervention_2_, and *t*(*time*−*T*_3_)*intervention_3_ were interaction terms between intervention and year, and β_3_, β_5_, andβ_7_, respectively, indicate the slope change following the intervention. *Fourier* terms consisting of sine/cosine three pairs were used to adjust seasonal variations. ε_*t*_ is the error term.

We conducted two further analyses to evaluate the intervention effects. First, models were stratified to investigate any differences in trends by gender, the urbanization rate (≤ 45.14 vs. >45.14 %), age group, and the number of pesticide vs. non-pesticide suicides. The urbanization rate in all surveillance sites was dichotomized at the median as the cutoff point (high-urbanization rate sites as urban or low-urbanization rate sites as rural) based on China's 2010 census data. Age was categorized into three groups (15–44, 45–64, and ≥65 years old). Suicides involving methods other than pesticides were calculated by subtracting suicides by pesticide from suicides by all other methods. Second, due to the potential lag effect of the intervention, we repeated the ITS analysis in suicide by pesticide using the time lags between 1 and 18 months after introducing the policy in December 2008, April 2012, and July 2016, respectively.

We calculated the rate ratio (RR), the monthly percent change (MPC), the average monthly percent change (AMPC), and its 95% confidence intervals (CIs) for the periods pre-intervention and post-intervention. The details are found in the Appendix. The deseasonalized curve was used to depict the trend of change in suicide. We checked for autocorrelation by the autocorrelation function and the partial autocorrelation function (Appendix Figure 3). The model-selection strategies in this study were the smallest Bayesian Information Criterion (BIC) and optimal interpretability. R 4.1.2 software (R Foundation for Statistical Computing, Vienna, Austria, https://www.R-project.org/) was used for all statistical analyses.

## 3. Results

### 3.1. Overall trends

The age-standardized suicide rate in China showed a downward trend from 2006 to 2018 declining by 45.1% from 12.70 in 2006 to 6.98 per 100,000 in 2018 (Table 2). Similarly, the age-standardized pesticide suicide rate decreased by 60.5% from 6.50 in 2006 to 2.56 per 100,000 in 2018 and suicides using methods other than pesticides declined by 28.9% from 6.20 to 4.41 per 100,000 (Table 2). From 2006 to 2018, the proportional contribution of pesticide poisoning to total suicides decreased from 51.2 to 36.8% (Table 2, Appendix Table 2). The lag effect test showed that the trend of suicides by pesticide was significantly changed and tended to steepen after interventions 1 and 3 lagged from 0 to 14 and from 0 to 18 months, respectively, and tended to flatten after intervention 2 lagged from 0 to 6 months (Appendix Figure 1).

**Table 2:** Yearly suicide rate and pesticide suicide rates in China, 2006–2018.

**Year**	**Suicide (per 100,000)**		**Pesticide (per 100,000)**		**Ratio of pesticide/suicide (%)**	
	**Standardized**	**Unstandardized**	**Standardized**	**Unstandardized**	**Standardized**	**Unstandardized**
2006	12.70	12.50	6.50	6.51	51.16	52.05
2007	12.69	12.55	6.55	6.54	51.63	52.11
2008	12.76	12.69	6.44	6.43	50.45	50.69
2009	11.90	11.86	6.10	6.11	51.31	51.48
2010	10.95	10.95	5.34	5.34	48.80	48.80
2011	9.98	10.11	4.94	5.00	49.50	49.41
2012	9.74	10.04	4.74	4.88	48.70	48.57
2013	9.42	9.85	4.61	4.80	48.91	48.72
2014	8.98	9.53	4.22	4.46	46.95	46.79
2015	8.71	9.35	4.09	4.36	46.90	46.63
2016	8.26	8.98	3.54	3.82	42.83	42.57
2017	7.55	8.23	3.12	3.39	41.28	41.22
2018	6.98	7.68	2.56	2.85	36.76	37.05

Before the policy intervention period in December 2008, suicide by pesticide and non-pesticide MPC was not statistically significant, but a significant decrease was evident after December 2008, and the decreasing trend significantly flattened after the policy in April 2012 (Figure 2, Table 3, Appendix Table 3). After July 2016, the decreasing trend of suicide by pesticide became significantly steeper (RR = 0.992, 95% CIs: 0.990–0.994), while the trend change in suicide by non-pesticide methods was not significant (RR = 0.999, 95% CIs: 0.997–1.001) (Figure 2, Table 3, Appendix Table 3).

**Table 3:** Slope and level changes for suicide, pesticide suicide, and non-pesticide suicide rate ratio before and after pesticide bans in December 2008, April 2012, and July 2016.

		**Pesticide suicide**	***P***	**Non-pesticide suicide**	***P***	**Suicide**	***P***
		**RR (95% CI)**		**RR (95% CI)**		**RR (95% CI)**	
**Nationwide**	Underlying linear time trend	0.999 (0.998 to 1.001)	0.397	1.001 (0.999 to 1.002)	0.393	1.000 (0.999 to 1.001)	0.713
	Level change at intervention 1	0.987 (0.950 to 1.025)	0.491	0.974 (0.935 to 1.015)	0.218	0.978 (0.952 to 1.005)	0.116
	Level change at intervention 2	1.075 (1.042 to 1.108)	< 0.001	1.006 (0.973 to 1.039)	0.740	1.038 (1.015 to 1.063)	0.001
	Level change at intervention 3	0.946 (0.910 to 0.982)	0.004	1.006 (0.966 to 1.049)	0.767	0.968 (0.942 to 0.995)	0.019
	Trend change after intervention 1	0.993 (0.991 to 0.994)	< 0.001	0.994 (0.993 to 0.996)	< 0.001	0.993 (0.992 to 0.994)	< 0.001
	Trend change after intervention 2	1.003 (1.002 to 1.004)	< 0.001	1.004 (1.003 to 1.005)	< 0.001	1.004 (1.003 to 1.004)	< 0.001
	Trend change after intervention 3	0.992 (0.990 to 0.994)	< 0.001	0.999 (0.997 to 1.001)	0.244	0.997 (0.996 to 0.998)	< 0.001
**Urbanization level**							
**High**	Underlying linear time trend	0.990 (0.985 to 0.995)	< 0.001	0.999 (0.997 to 1.001)	0.260	0.995 (0.993 to 0.997)	< 0.001
	Level change at intervention 1	1.132 (0.989 to 1.296)	0.072	1.243 (1.123 to 1.375)	< 0.001	1.089 (1.033 to 1.149)	0.002
	Level change at intervention 2	1.148 (1.015 to 1.298)	0.028	1.062 (1.006 to 1.120)	0.029	1.116 (1.065 to 1.168)	< 0.001
	Level change at intervention 3	0.865 (0.758 to 0.988)	0.032	0.966 (0.909 to 1.027)	0.267	0.937 (0.889 to 0.987)	0.014
	Trend change after intervention 1	0.999 (0.993 to 1.005)	0.722	0.995 (0.992 to 0.997)	< 0.001	0.996 (0.994 to 0.998)	0.001
	Trend change after intervention 2	1.006 (1.001 to 1.011)	0.012	1.007 (1.005 to 1.009)	< 0.001	1.007 (1.006 to 1.009)	< 0.001
	Trend change after intervention 3	0.993 (0.987 to 1.000)	0.047	1.000 (0.997 to 1.003)	0.834	0.999 (0.997 to 1.002)	0.578
**Low**	Underlying linear time trend	1.004 (1.002 to 1.005)	< 0.001	1.002 (0.998 to 1.005)	0.314	1.003 (1.002 to 1.004)	< 0.001
	Level change at intervention 1	0.916 (0.884 to 0.948)	< 0.001	0.923 (0.845 to 1.007)	0.072	0.916 (0.894 to 0.938)	< 0.001
	Level change at intervention 2	1.024 (0.997 to 1.052)	0.086	0.971 (0.898 to 1.051)	0.467	0.999 (0.982 to 1.015)	0.879
	Level change at intervention 3	0.969 (0.936 to 1.003)	0.071	1.016 (0.933 to 1.107)	0.711	1.004 (0.979 to 1.029)	0.757
	Trend change after intervention 1	0.990 (0.989 to 0.991)	< 0.001	0.994 (0.990 to 0.998)	0.003	0.991 (0.991 to 0.992)	< 0.001
	Trend change after intervention 2	1.001 (1.000 to 1.002)	0.013	1.001 (0.999 to 1.004)	0.326	1.001 (1.001 to 1.002)	< 0.001
	Trend change after intervention 3	0.991 (0.989 to 0.993)	< 0.001	0.998 (0.994 to 1.003)	0.441	0.994 (0.993 to 0.996)	< 0.001
**Sex**							
**Male**	Underlying linear time trend	1.000 (0.999 to 1.001)	0.885	1.000 (0.997 to 1.003)	0.870	1.000 (1.000 to 1.001)	0.203
	Level change at intervention 1	0.955 (0.926 to 0.984)	0.003	0.985 (0.910 to 1.067)	0.713	0.980 (0.962 to 0.998)	0.028
	Level change at intervention 2	1.048 (1.024 to 1.073)	< 0.001	1.020 (0.950 to 1.094)	0.593	1.052 (1.038 to 1.066)	< 0.001
	Level change at intervention 3	0.919 (0.891 to 0.947)	< 0.001	0.985 (0.911 to 1.065)	0.696	0.980 (0.962 to 0.998)	0.034
	Trend change after intervention 1	0.994 (0.993 to 0.995)	< 0.001	0.995 (0.991 to 0.998)	0.002	0.993 (0.993 to 0.994)	< 0.001
	Trend change after intervention 2	1.003 (1.002 to 1.004)	< 0.001	1.005 (1.002 to 1.007)	0.001	1.004 (1.004 to 1.004)	< 0.001
	Trend change after intervention 3	0.990 (0.988 to 0.991)	< 0.001	0.999 (0.995 to 1.003)	0.694	0.995 (0.994 to 0.996)	< 0.001
**Female**	Underlying linear time trend	0.999 (0.997 to 1.001)	0.218	1.001 (0.997 to 1.004)	0.706	1.000 (0.998 to 1.001)	0.743
	Level change at intervention 1	1.027 (0.978 to 1.078)	0.281	0.912 (0.825 to 1.009)	0.074	0.982 (0.943 to 1.023)	0.375
	Level change at intervention 2	1.109 (1.066 to 1.154)	< 0.001	0.948 (0.865 to 1.039)	0.252	1.028 (0.993 to 1.065)	0.121
	Level change at intervention 3	0.972 (0.927 to 1.020)	0.254	0.949 (0.860 to 1.047)	0.295	0.964 (0.926 to 1.003)	0.068
	Trend change after intervention 1	0.991 (0.989 to 0.993)	< 0.001	0.995 (0.991 to 1.000)	0.056	0.993 (0.991 to 0.995)	< 0.001
	Trend change after intervention 2	1.003 (1.001 to 1.004)	< 0.001	1.003 (0.999 to 1.006)	0.174	1.003 (1.002 to 1.004)	< 0.001
	Trend change after intervention 3	0.994 (0.992 to 0.997)	< 0.001	1.001 (0.996 to 1.006)	0.719	0.998 (0.997 to 1.000)	0.110
**Age group (years)**							
**15–44**	Underlying linear time trend	0.999 (0.996 to 1.001)	0.330	1.003 (1.002 to 1.004)	< 0.001	1.000 (0.999 to 1.002)	0.531
	Level change at intervention 1	0.988 (0.919 to 1.061)	0.734	0.926 (0.899 to 0.953)	< 0.001	0.962 (0.928 to 0.996)	0.030
	Level change at intervention 2	1.083 (1.023 to 1.146)	0.006	1.018 (0.995 to 1.042)	0.125	1.054 (1.028 to 1.081)	< 0.001
	Level change at intervention 3	1.048 (0.975 to 1.126)	0.203	1.009 (0.980 to 1.038)	0.561	1.052 (1.014 to 1.091)	0.007
	Trend change after intervention 1	0.993 (0.990 to 0.995)	< 0.001	0.992 (0.991 to 0.993)	< 0.001	0.993 (0.992 to 0.994)	< 0.001
	Trend change after intervention 2	1.000 (0.998 to 1.002)	0.970	1.004 (1.003 to 1.004)	< 0.001	1.002 (1.001 to 1.003)	< 0.001
	Trend change after intervention 3	0.989 (0.986 to 0.993)	< 0.001	1.007 (1.006 to 1.009)	< 0.001	1.000 (0.998 to 1.002)	0.995
**45–64**	Underlying linear time trend	1.000 (0.999 to 1.002)	0.669	1.003 (0.999 to 1.006)	0.139	1.002 (1.000 to 1.004)	0.074
	Level change at intervention 1	0.960 (0.926 to 0.995)	0.027	0.930 (0.841 to 1.029)	0.162	0.949 (0.900 to 1.000)	0.051
	Level change at intervention 2	1.074 (1.046 to 1.103)	< 0.001	0.955 (0.873 to 1.045)	0.317	1.019 (0.975 to 1.065)	0.399
	Level change at intervention 3	0.923 (0.890 to 0.957)	< 0.001	0.978 (0.886 to 1.079)	0.656	0.978 (0.929 to 1.030)	0.405
	Trend change after intervention 1	0.993 (0.991 to 0.994)	< 0.001	0.994 (0.990 to 0.999)	0.013	0.993 (0.991 to 0.995)	< 0.001
	Trend change after intervention 2	1.003 (1.002 to 1.004)	< 0.001	1.003 (1.000 to 1.007)	0.053	1.003 (1.002 to 1.005)	< 0.001
	Trend change after intervention 3	0.990 (0.988 to 0.992)	< 0.001	0.995 (0.990 to 1.000)	0.040	0.993 (0.990 to 0.995)	< 0.001
**≥65**	Underlying linear time trend	0.999 (0.998 to 1.000)	0.128	0.998 (0.994 to 1.002)	0.349	0.998 (0.997 to 0.999)	0.001
	Level change at intervention 1	1.019 (0.991 to 1.049)	0.187	0.974 (0.869 to 1.093)	0.656	1.027 (1.000 to 1.055)	0.052
	Level change at intervention 2	1.078 (1.060 to 1.097)	< 0.001	0.989 (0.891 to 1.097)	0.832	1.052 (1.031 to 1.073)	< 0.001
	Level change at intervention 3	0.872 (0.847 to 0.898)	< 0.001	0.958 (0.856 to 1.072)	0.455	0.936 (0.911 to 0.962)	< 0.001
	Trend change after intervention 1	0.992 (0.991 to 0.993)	< 0.001	0.997 (0.991 to 1.002)	0.211	0.994 (0.993 to 0.995)	< 0.001
	Trend change after intervention 2	1.005 (1.005 to 1.006)	< 0.001	1.004 (1.000 to 1.008)	0.075	1.005 (1.004 to 1.006)	< 0.001
	Trend change after intervention 3	0.996 (0.994 to 0.997)	< 0.001	0.996 (0.991 to 1.002)	0.200	0.996 (0.995 to 0.998)	< 0.001

RR, rate ratio; CIs, confidence intervals. Intervention 1: banned five highly hazardous organophosphorus (HHOP) pesticides implemented in December 2008. Intervention 2: stopped the new registration and production of paraquat parent drug and aqueous solutions and regulated the existing paraquat production and after-sales services in April 2012. Intervention 3: stopped domestic sales and use of paraquat aqueous solution in July 2016.

### 3.2. Trends by sex

**Figure 1:**
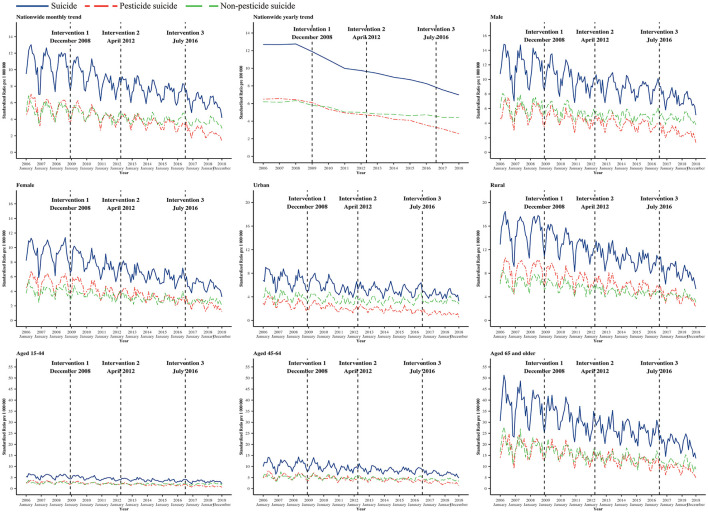
Trend of monthly suicide, pesticide suicide, and non-pesticide suicide rate in China from January 2006 to December 2018. Intervention 1: banned five highly hazardous organophosphorus (HHOP) pesticides implemented in December 2008. Intervention 2: stopped the new registration and production of paraquat parent drug and aqueous solutions and regulated the existing paraquat production and after-sales services in April 2012. Intervention 3: stopped domestic sales and use of paraquat aqueous solution in July 2016.

**Figure 2:**
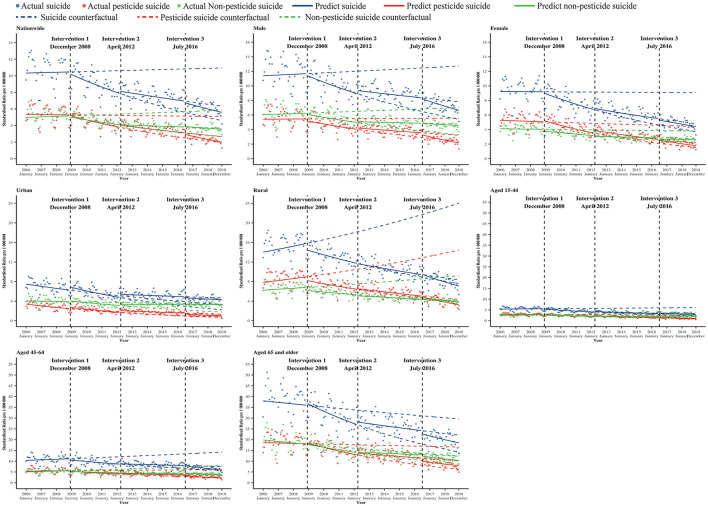
The isolated trend cycle curve was smoothed of monthly suicide, pesticide suicide, and non-pesticide suicide rate after excluding the seasonal variations, including the counterfactual in China from January 2006 to December 2018. Intervention 1: banned five highly hazardous organophosphorus (HHOP) pesticides implemented in December 2008. Intervention 2: stopped the new registration and production of paraquat parent drug and aqueous solutions and regulated the existing paraquat production and after-sales services in April 2012. Intervention 3: stopped domestic sales and use of paraquat aqueous solution in July 2016.

The standardized suicide rate for male and female individuals decreased by 40.6 and 50.8% from 14.15 and 11.22 per 100,000 in 2006 to 8.41 and 5.53 per 100,000 in 2018. At the same time, the rate of suicide by pesticide for male and female individuals decreased by 57.7 and 63.5%, respectively, from 2006 to 2018, from 6.66 to 2.82 per 100,000 for male individuals and from 6.33 to 2.31 per 100,000. Before the policy intervention (December 2008), the MPC in suicide by pesticides among male and female individuals was not statistically significant, but a significant decrease (male individuals: MPC = −0.6, 95% CIs: −0.8 to −0.5; female individuals: MPC = −1.0, 95% CIs: −1.2 to −0.8) was evident after December 2008 (male individuals: RR = 0.994, 95% CIs: 0.993–0.995; female individuals: RR = 0.991, 95% CIs: 0.989–0.993). The trend of suicide by pesticide tended to be flat and was significant in both male and female individuals after the intervention in April 2012 (male individuals: RR = 1.003, 95% CIs: 1.002–1.004; female individuals: RR = 1.003, 95% CIs: 1.001–1.004). After the intervention in July 2016, suicide by pesticide fell significantly in both male individuals and female individuals (male individuals: RR = 0.990, 95% CIs: 0.988–0.991; female individuals: RR = 0.994, 95% CIs: 0.992–0.997). However, the trend change in suicide by the non-pesticide method was significant only for male individuals, with a significant decrease after the intervention in December 2008 and a tendency to slow down after April 2012 (Table 3, Figures 1, 2, Appendix Table 3).

### 3.3. Trends by age group

The standardized rate of suicide among those aged 15–44, 45–65, and ≥65 years declined by 43.5, 40.9, and 49.7%, respectively, from 6.68, 13.01, and 45.80 per 100,000 in 2006 to 3.78, 7.70, and 23.03 per 100,000 in 2018. At the same time, the rate of suicide by pesticide for people aged 15–44, 45–64, and ≥65 years also declined by 68.1, 57.4, and 56.4%, from 3.55, 6.83, and 22.23 per 100,000 in 2006 to 1.13, 2.91, and 9.69 per 100,000 in 2018. Before the policy intervention in December 2008, the changes in the rate of suicide by pesticide, the non-pesticide suicide rate, and the suicide rate in all age groups were not statistically significant. However, after the policy intervention in December 2008, the changes in the rate of suicide by pesticides for all age groups were statistically significant, with MPC −0.9 (95% CIs: −1.1 to −0.6), −0.7 (95% CIs: −0.8 to −0.6), and −0.9 (95% CIs: −1.0 to −0.8), respectively. After the intervention in April 2012, the change was significant in those aged 45–64 years and aged 65 years and older, and the trend tended to be flat (aged 15–44 years: RR=1.000, 95% CIs: 0.998–1.002; aged 45–64 years: RR = 1.003, 95% CIs: 1.002 to 1.004; aged 65 years and older: RR = 1.005, 95% CIs: 1.005 to 1.006). After the intervention in July 2016, the trend was decreasing and significant in all age groups. Meanwhile, the non-pesticide suicide rate significantly decreased in those aged 15–44 and 45–64 years after the intervention in December 2008, the trend change tended to flat significant only in those aged 15–44 years after the intervention in April 2012 and July 2016. All changes in suicide by non-pesticide for those aged 65 years and older were not significant after interventions 1, 2, and 3 (Table 3, Figures 1, 2, Appendix Table 3).

### 3.4. Trends by the urbanization level

The age-standardized rate in areas of low urbanization decreased by 48.6% from 17.68 per 100,000 in 2006 to 9.08 per 100,000 in 2018. Age-standardized rates decreased by 39.4% from 8.77 per 100,000 in 2006 to 5.31 per 100,000 in 2018 in areas of high urbanization. The rate of suicide by pesticides in low- and high-urbanization rate areas declined by 57.2 and 67.3%, respectively, from 9.82 and 3.87 per 100,000 in 2006 to 4.21 and 1.27 per 100,000 in 2018. Before the policy intervention (December 2008), the underlying trend showed a significant decrease with MPC of −1.0 (95% CIs: −1.5 to −0.5) in the pesticide suicide rate in high-urbanization areas (RR = 0.990, 95% CIs: 0.985–0.995), a significant increase with MPC of 0.4 (95% CIs: 0.2–0.5) in the pesticide suicide rate in low-urbanization areas (RR = 1.004, 95% CIs: 1.002–1.005), and no significant change in non-pesticide rate in high- and low-urbanization areas. In contrast, following the intervention in December 2008, a decrease in the rate of suicide by pesticide was evident in low-urbanization areas (RR = 0.990, 95% CIs: 0.989–0.991). After the intervention in April 2012, there was no change in the trend of suicide by pesticide change in low-urbanization areas but increased in high-urbanization areas (high-urbanization areas: RR = 1.006, 95% CIs: 1.001–1.011; low-urbanization areas: RR = 1.001, 95% CIs: 1.000–1.002). After the intervention in July 2016, the trend changed significantly and fell more steeply in low-urbanization areas (high-urbanization areas: RR = 0.993, 95% CIs: 0.987–1.000; low-urbanization areas: RR = 0.991, 95% CIs: 0.989–0.993). During the same period, the underlying trend change in suicide by non-pesticide was not significant. The decreasing trend tended to be steeper in high- and low-urbanization areas only after the intervention in December 2008, and the decreasing trend tended to be flattened in high-urbanization areas after the intervention in April 2012 (Table 3, Figures 1, 2, Appendix Table 3).

## 4. Discussion

This study showed that China's regulations of banning and restricting pesticides were associated with significant contemporaneous declines in total suicide from 2006 to 2018, largely due to reductions in suicide by pesticide poisoning. A decline in total suicide and suicide by pesticide was evident in the period after the policy intervention in December 2008 (when HHOP pesticides were fully banned rather than restricted in use), with a flat decreasing trend after the policy intervention in April 2012 (when the new registration and production of paraquat parent drug and aqueous solution stopped and the existing paraquat production and after-sales services were regulated), and a steep decreasing trend after the policy intervention in July 2016 (when paraquat aqueous solutions were entirely banned rather than restricted in use). The decreases were more prominent in people living in urban areas, among female individuals, and people aged 15–44 years. Meanwhile, a lagged analysis suggests an increase in the association after the implementation of China's pesticide regulations in July 2016.

A study from South Korea also showed that the impact of a pesticide (paraquat) ban was more significant for both men and the elderly on suicide prevention and control, but the study only observed the short-term association over a 2-year follow-up period (19). Qin et al. conducted research to explore the suicide rate change trend in Inner Mongolia of China between 2008 and 2015 (25). The findings showed that suicide rates were higher in 2008–2011 than in 2012–2015, a period prior to the implementation of pesticide regulations in this region in 2011 (25). Chang et al. evaluated the trends of suicide by pesticide poisoning before and after paraquat ban implementation in Taiwan from 2011 to 2019 (39). The result showed that pesticide and paraquat suicides in the whole population substantially reduced with the paraquat ban implementation. Jiang et al. (40) found that, during the period 2002 to 2015, there is a significant drop in suicide rates in China. However, the overall suicide rate showed a slower pace after 2006 (40). Liu et al. concentrated on the trends of the suicide rate and the pesticide self-poisoning suicide rate in rural China from 2009 to 2014 (41). Given the inconsistent time periods and locations of these several studies, we were unable to compare the trend and speed of the national suicide rate decline. The current study extends this regional study (25, 39) and is based on an assessment of national pesticide regulations over a longer period and evaluates whether there were any contemporaneous declines in suicide over a much longer period and the first-time studied paraquat pesticide policies' effect on suicide in mainland China.

Similar to the research performed in other countries (42, 43), the obvious seasonality of suicide and pesticide suicide in China was present in our study, with the suicide rate peaking in spring and declining in winter (43), although seasonality tended to diminish (44). Between 2006 and 2018, the rate of suicide by pesticides in China declined at an average annual rate of 4.7%, with 4.4, 4.4, and 5.2% for male individuals, people living in rural areas, and people aged 15–44 years, respectively. At present, the suicide rate of male individuals in China is still higher than that of female individuals and remains much higher in rural areas than in urban areas (14). Considering that the suicide rate in China has declined dramatically over the past for more than a decade and the proportion of suicide by pesticides dropped from over half to approximately a third, we consider the pesticide ban and restriction policy to have been implemented effectively in reducing the burden of suicide.

Our findings suggest that the potential impact of pesticide prohibition and restriction on suicide by pesticide was greater in rural areas. Simultaneously, the reduction in suicide by the non-pesticide method implies that the trend change in this period may be attributed to natural variation or other non-pesticide national suicide intervention policies, but the natural variation assumption can be discredited by comparing urban areas with rural areas and male suicide changes with female suicide changes. Moreover, the trend showed a different shift in the suicide and non-pesticide suicide groups. The association can be observed after the policy of banning and restricting pesticides was introduced intensively after December 2008, as the intervention point, when the five important HHOPs (methamidophos, monocrotophos, methyl parathion, parathion, and ammonium phosphate) were wholly removed from the market in China (45). Before 2008, the prohibition and restriction policies on these high-toxicity pesticides had focused on revoking the relevant certificates of production, sale, and use (46, 47), which implies that it was only in 2008 that these organophosphorus pesticides were removed entirely from the Chinese market. Therefore, it is likely that the policy of banning and restricting the sale and use of pesticides had lagged impacts on the prevention of suicide by pesticides in the period post-2008, which was consistent with the stronger association evident in the lagged analysis in this current study.

Our results showed an unlikely effect of the regulated paraquat-related product in April 2012. The trend of suicide by pesticide tended to slow down significantly. Compared with the non-pesticide suicide group, a similar trend changed in both groups, prompting us to infer that a risk factor of suicide flattened the decrease of suicide, and even in rural areas where the intervention effect was more evident, the policy restriction effect may be masked by the risk factor. China has the largest periodically floating population of individuals migrating from rural to urban regions, who tend to have low socioeconomic and health status (48). The inter-provincial migrants increased to the highest level in 2012 and faced more life challenges than others (49, 50). We hypothesize that the social phenomenon of migration influenced suicide and suicide by pesticides in China, particularly in 2012. However, stopping domestic sales and the use of paraquat aqueous solutions in July 2016 effectively decreased suicide by pesticide.

China is the world's largest pesticide user, is the second-largest pesticide producer, and has the largest agricultural population. Many suicides in China are believed to be impulsive as a reaction to acute psychological distress (13); the availability of highly hazardous pesticides facilitates such impulsive suicides (making low-intent acts of self-poisoning lethal). Research from other countries indicates regulations banning highly toxic pesticide regulations can lead to falls in method-specific and, in some cases (e.g., Sri Lanka, Bangladesh, South Korea, and China Taiwan), overall suicides (39, 51). Page et al. analyzed the national suicide mortality trends of China from 2006 to 2013 and found that hanging, as a proportion of all suicides, increased from 27 to 31%, while the contribution of pesticide poisoning to total suicides declined from 55 to 49%, although the absolute numbers of suicides using both methods reduced substantially (9). The current study also found that the proportion of suicide by pesticide has decreased in recent decades. The results of this study are consistent with those from previous studies and further provide evidence for timely and effective intervention in low- and middle-income countries where suicide by pesticide remains the main method of suicide.

In order to improve the quality and safety of agricultural products and reduce environmental pollution, the production, use, and trade of high-toxic and high-risk pesticides is increasingly controlled worldwide. The formulation and implementation of pesticide bans and policies of restriction is a process of multi-sectoral integration and cooperation with relevant stakeholders. This multi-sectoral collaboration is indispensable in the context of suicide prevention. Since the Stockholm Convention on Persistent Organic Pollutants (POPs) was signed by the Chinese delegation comprising representatives from the Ministry of Health, the Ministry of Agriculture, the State Economic and Trade Commission, and the General Administration of Environmental Protection in 2002, China's pesticide ban and restriction has been much strengthened, institutionalized, and standardized (45, 52). With the introduction of a series of prohibition and restriction policies, the Ministry of Agriculture has intensified the research and development of low-toxic biological pesticides.

One key strength of this study was the use of a 15-year period of monthly suicide data collected by the DSP system, a continuous and consistent source of data that included certification of death with relevant local departments (civil affairs, public security, and the maternal and child healthcare sector) (26). This strongly supports the accessibility, comprehensiveness, and quality of suicide information in China (27). Another strength of our study concerns the comprehensive analyses of the effect on multiple key pesticide policies to avoid pesticide suicide decrease misattributed to concerned ban but neglected potential policy or elusive factor effects; thus, we further discussed the pesticides policies on suicide and non-pesticide suicide. Additionally, an ITS analysis was designed to evaluate the trends of population-level intervention (34), including at various lag periods, which accounted for seasonality and autocorrelation in time series.

This study had some limitations. First, similar to all other surveillance systems worldwide, the problem of accuracy and completeness is inevitable in the DSP system, where the potential sources of system bias remain. The surveillance data included in the analysis also did not adjust for potential under-reporting of suicide, which may cause information bias (11). However, its impact on trend analysis would be small. Second, the primary purpose of this study was to evaluate the single pesticide policy effect, but multiple policy efforts to ban different pesticides occurred at different time points during 2006–2021. Despite the substantial evidence collected, the performance of these policies and the effect between these policies could not be definitely determined. Furthermore, the multiple interventions also make it challenging to define the intervention time points in the interrupted time series regression analysis. Our approach to define the intervention time points was informed by the change point detection method implemented by segmented regression, and this may potentially lead to false positive findings in the interrupted time series regression analysis, although our final decision of the intervention time points was based on an analysis of the content of the pesticide ban policies. Third, only 2 years of suicide data are available before the HHOP pesticides ban in 2008 in our analyses, even though no specific number of time points was demanded, a short pre-intervention period may raise the uncertainty in the post-intervention trend judgment (34, 36), but the monthly data would make up for this deficiency, to a certain extent. Fourth, ITS analysis is a quasi-experimental design method that assumes the omitted variable effect consistent across the study period; however, the facts may be different (12).

## 5. Conclusion

Recent declines in suicide in China occurred contemporaneously with regulatory bans and restrictions on five key HHOPS in 2008 and a paraquat aqueous solution ban in 2016. Similar declines were observed for suicide by pesticide poisoning and for suicides using other methods since 2008. Larger declines were observed for suicide by pesticide poisoning than that for suicides using other methods, indicating the potential influence of pesticide bans on pesticide suicide trends since 2008.

## Data availability statement

The raw data supporting the conclusions of this article will be made available by the authors, without undue reservation.

## Author contributions

SL designed and supervised the study. YY, YJ, and RL analyzed the data and wrote the first draft. All authors have interpreted the data and contributed to intellectual content.
