# Supplementary material for: Impact of pesticide regulations on mortality from suicide by pesticide in China: an interrupted time series analysis
Source: Front Psychiatry. 2023 Sep 4;14:1189923. doi: 10.3389/fpsyt.2023.1189923 (PMC10507350; doi:10.3389/fpsyt.2023.1189923)
Supplement: Supplementary file 1 [file Data_Sheet_1.PDF]

Supplementary Online Content

Outline

Statistical method

Appendix Table 1. The breakpoint location by change point detection (CPD)

Appendix Table 2. Average suicide and suicide by pesticide standardized number in surveillance points in different periods

Appendix Table 3. The monthly change percentage for standardized suicide, pesticide suicide and non-pesticide suicide rate in different periods

Appendix Table 4. Impact of pesticide policies on the standardized number of suicide by pesticide in surveillance points (MAS)

Appendix Figure 1. The pesticide suicide trend of changes after the intervention effect lagged in China

Appendix Figure 2. The trend of monthly suicide, pesticide suicide and non-pesticide suicide rate and corresponding counterfactual in China from Jan 2006 to Dec 2018

Appendix Figure 3. The model residual autocorrelation and partial autocorrelation test before and after the model adjusted

Statistical method

The intervention points were defined by combined change point detection (CPD) and content analysis of pesticide policy. We used CPD to explore any trend change in suicide by pesticide. CPD iterative procedure implemented by *segmented* package in R,[1,2] and respectively explored the monthly location of suicide by pesticides breakpoints that we assumed 1-4 breakpoints existed in 2006-2018 (appendix table 1). The one breakpoint situation implication only one effective intervention around 2016, but the intervention in December 2008 and China banned the use of methamidophos, monocrotophos, methyl parathion, parathion and ammonium phosphate five important OP HHPs we can not ignore.[3] When we analysed the two breakpoints situation, the wide confidence interval may implicate more than one breakpoint in the interval. Thus, we considered three breakpoints and compared them with four breakpoints results, finding four breakpoints overlap two intervals at different times, respectively. Finally, we selected the three breakpoints situation and considered the policy implementation, and the potentially effective policies including banning HHOP in December 2008, stopping registration and production of new paraquat parent drug and aqueous solutions, and simultaneously regulating the existing paraquat production and after-sales services in April 2012, and stopping domestic sales and use of paraquat aqueous solution in July 2016, respectively. (table 1).

Initially, the negative binomial regression was used for the ITS to evaluate pesticide policies in China. However, the autocorrelation function and partial autocorrelation function tested the residual in the negative binomial regression and showed obvious autocorrelation even though we adjusted by adding Fourier term or spline function in regression.[4,5] Finally, we fitted the model using the generalized least squares method which allows autocorrelation in the data.[6]

In our study, several indicators were used to describe the changing trend of suicide and suicide by pesticide,[7] and the detail above shows below:

Model equation:

$$log(S_t) = \beta_0 + offset\big(log(people_t)\big) + \beta_1 time + \beta_2 intervention_1 + \beta_3 (time - T_1) * intervention_1 + \beta_4 intervention_2 + \beta_5 (time - T_2) * intervention_2 + \beta_6 intervention_3 + \beta_7 (time - T_3) * intervention_3 + \beta_{8-13} fourier + \varepsilon_t$$

The Rate Ratio (RR) indicates the suicide or suicide by pesticide rate changes with the analysis factor change, and we compute the RR of level change and trend change independently.

RR= $rate_t / rate_{t-1}$=$(S_t / people_t)/(S_{t-1}/people_{t-1})$

$rate_t$ represents standardized suicide or suicide by pesticide rate in time *t*. In the regression model, we used standardized suicide and population number. Thus

$people_t = people_{t-1}$

RR=S_t / S_{t-1}=$\frac{e^{\beta t}}{e^{\beta(t-1)}} = e^{\beta}$

The confidence intervals (CI) of RR compute by $e^{\beta \pm Se_{\beta} Z_{1-\alpha/2}}$

1. The Monthly Percent Change (MPC) indicates the monthly change percentage in suicide or suicide by pesticide in the segment.

   MPC=$(rate_t - rate_{t-1})/rate_{t-1} \times 100$=$(S_t - S_{t-1})/S_{t-1} \times 100$

   =$(e^{\beta_x t} - e^{\beta_x (t-1)})/e^{\beta_x (t-1)} \times 100$=$(e^{\beta_x} - 1)\times 100$

   The CI of MPC compute by $(e^{\beta_x \pm Se_{\beta_x} Z_{1-\alpha/2}} - 1) \times 100.$

2. The Average Monthly Percent Change (AMPC) indicates the monthly change percentage in suicide or suicide by pesticide in the whole study interval.

   AMPC=$(e^{\Sigma(l_x \beta_x)/N} - 1)\times 100$

   l_x is the length of a segment. The CI of AMPC compute by

   $(e^{\Sigma(l_x \beta_x)/N \pm \sqrt{\Sigma(\left(\frac{l_x}{N}\right)^2 Se_{\beta_x}^2)} Z_{1-\alpha/2}} - 1) \times 100,$ *N* is all time point number.

3. The Mortality Attributable to Suicide (MAS) indicate reduced suicide or suicide by pesticide number may be attributed to pesticide ban in time *t*.

   MAS=$\widehat{sc}_t - \hat{s}_t$

   $\widehat{sc}_t$ is predicted monthly suicide number without pesticide policy, and performed by counterfactual, $\hat{s}_t$ is predicted monthly suicide number with pesticide policy.

References

1. Muggeo VM. Estimating regression models with unknown break-points. *Stat Med* 2003; **22**(19): 3055-71.

2. Muggeo VM. Segmented: an R package to fit regression models with broken-line relationships. *R news* 2008; **8(1)**: 20-5.

3. Liu JG, Hu JX, Tang XY. Preliminary identification of China's basic obligations and obstacles in fulfilling the Stockholm Convention. *Legal System and Management* 2002; **8**: 6-9,23.

4. Bernal JL, Cummins S, Gasparrini A. Interrupted time series regression for the evaluation of public health interventions: a tutorial. *Int J Epidemiol* 2017; **46**(1): 348-55.

5. Bhaskaran K, Gasparrini A, Hajat S, Smeeth L, Armstrong B. Time series regression studies in environmental epidemiology. *Int J Epidemiol* 2013; **42**(4): 1187-95.

6. Dayer MJ, Jones S, Prendergast B, Baddour LM, Lockhart PB, Thornhill MH. Incidence of infective endocarditis in England, 2000-13: a secular trend, interrupted time-series analysis. *Lancet* 2015; **385**(9974): 1219-28.

7. Statistical Methodology and Applications Branch SRP. Joinpoint Regression Program, Version 4.9.1.0: National Cancer Institute; 2022.

Appendix Table 1. The breakpoint location in time elapses by change point detection (CPD)

Number of breakpoints	Location of the month (95% CI)
**One**	125.00 (111.11 to 138.89)
**Two**	30.00 (-4.03 to 64.03)
	125.14 (108.87 to 141.42)
**Three**	41.96 (26.90 to 57.02)
	60.00 (41.17 to 78.83)
	115.02 (100.44 to 129.60)
**Four**	54.99 (48.08 to 61.91)
	59.00 (48.70 to 69.30)
	116.53 (97.76 to 135.30)
	118.86 (81.48 to 156.23)

CI: confidence intervals

Appendix Table 2. Average suicide and suicide by pesticide standardized number in surveillance points in different periods

Period	Policies	Number of months	Average of suicide number (per year)	Average of suicide by pesticide number (per year)
January 2006 to December 2008	No policies	35	8768	4483
December 2008 to April 2012	Intervention 1	40	7395	3674
April 2012 to July 2016	Intervention 1/2	51	6252	2963
July 2016 to December 2018	Intervention 1/2/3	30	5015	1999

Intervention 1: banned five highly hazardous organophosphorus (HHOP) pesticides implemented in December 2008. Intervention 2: stopped the new registration and production of paraquat parent drug and aqueous solutions and regulated the existing paraquat production and after-sales services in April 2012. Intervention 3: stopped domestic sales and use of paraquat aqueous solution in July 2016.

Appendix Table 3. The monthly change percentage for standardized suicide, pesticide suicide and non-pesticide suicide rate in different periods

	Polices	Pesticide suicide		Non-pesticide suicide		Suicide	
		MPC (95% CI)	AMPC (95% CI)	MPC (95% CI)	AMPC (95% CI)	MPC (95% CI)	AMPC (95% CI)
**Nationwide**	No policies	-0.1 (-0.2 to 0.1)	-0.6 (-0.7 to -0.6)	0.1 (-0.1 to 0.2)	-0.2 (-0.3 to -0.1)	0 (-0.1 to 0.1)	-0.4 (-0.4 to -0.3)
	Intervention 1	-0.8 (-0.9 to -0.7)		-0.5 (-0.7 to -0.4)		-0.7 (-0.8 to -0.6)	
	Intervention 1/2	-0.5 (-0.6 to -0.4)		-0.1 (-0.2 to 0)		-0.3 (-0.4 to -0.2)	
	Intervention 1/2/3	-1.3 (-1.5 to -1.2)		-0.2 (-0.4 to 0)		-0.6 (-0.8 to -0.5)	
**Urbanization rate**							
**High**	No policies	-1.0 (-1.5 to -0.5)	-0.9 (-1.2 to -0.6)	-0.1 (-0.3 to 0.1)	-0.2 (-0.3 to 0)	-0.5 (-0.7 to -0.3)	-0.4 (-0.5 to -0.3)
	Intervention 1	-1.1 (-1.7 to -0.5)		-0.6 (-0.9 to -0.4)		-0.9 (-1.1 to -0.7)	
	Intervention 1/2	-0.5 (-1.0 to 0)		0.1 (-0.1 to 0.2)		-0.2 (-0.3 to 0)	
	Intervention 1/2/3	-1.2 (-1.8 to -0.5)		0.1 (-0.2 to 0.4)		-0.2 (-0.5 to 0)	
**Low**	No policies	0.4 (0.2 to 0.5)	-0.5 (-0.6 to -0.5)	0.2 (-0.2 to 0.5)	-0.3 (-0.4 to -0.1)	0.3 (0.2 to 0.4)	-0.4 (-0.4 to -0.3)
	Intervention 1	-0.6 (-0.8 to -0.5)		-0.4 (-0.8 to 0)		-0.5 (-0.6 to -0.5)	
	Intervention 1/2	-0.5 (-0.6 to -0.4)		-0.3 (-0.6 to 0)		-0.4 (-0.5 to -0.4)	
	Intervention 1/2/3	-1.4 (-1.6 to -1.2)		-0.4 (-0.9 to 0)		-1.0 (-1.1 to -0.9)	
**Sex**							
**Male**	No policies	0 (-0.1 to 0.1)	-0.5 (-0.6 to -0.5)	0 (-0.3 to 0.3)	-0.2 (-0.3 to 0)	0 (0 to 0.1)	-0.4 (-0.4 to -0.3)
	Intervention 1	-0.6 (-0.8 to -0.5)		-0.5 (-0.9 to -0.2)		-0.6 (-0.7 to -0.6)	
	Intervention 1/2	-0.3 (-0.4 to -0.3)		-0.1 (-0.3 to 0.2)		-0.2 (-0.3 to -0.2)	
	Intervention 1/2/3	-1.4 (-1.5 to -1.2)		-0.1 (-0.5 to 0.2)		-0.7 (-0.8 to -0.6)	
**Female**	No policies	-0.1 (-0.3 to 0.1)	-0.8 (-0.9 to -0.7)	0.1 (-0.3 to 0.4)	-0.1 (-0.3 to 0.1)	0 (-0.2 to 0.1)	-0.5 (-0.5 to -0.4)
	Intervention 1	-1.0 (-1.2 to -0.8)		-0.4 (-0.8 to 0.1)		-0.7 (-0.9 to -0.6)	
	Intervention 1/2	-0.7 (-0.9 to -0.6)		-0.1 (-0.5 to 0.2)		-0.4 (-0.6 to -0.3)	
	Intervention 1/2/3	-1.3 (-1.5 to -1.1)		0 (-0.5 to 0.5)		-0.6 (-0.8 to -0.4)	
**Age group (years)**							
**15-44**	No policies	-0.1 (-0.4 to 0.1)	-0.9 (-1.0 to -0.8)	0.3 (0.2 to 0.4)	0 (-0.1 to 0)	0 (-0.1 to 0.2)	-0.4 (-0.5 to -0.4)
	Intervention 1	-0.9 (-1.1 to -0.6)		-0.5 (-0.6 to -0.4)		-0.7 (-0.8 to -0.6)	
	Intervention 1/2	-0.8 (-1.0 to -0.7)		-0.2 (-0.2 to -0.1)		-0.5 (-0.6 to -0.4)	

	Intervention 1/2/3	-1.9 (-2.2 to -1.6)		0.6 (0.4 to 0.7)		-0.5 (-0.7 to -0.3)	
**45-64**	No policies	0 (-0.1 to 0.2)	-0.6 (-0.6 to -0.5)	0.3 (-0.1 to 0.6)	-0.1 (-0.3 to 0.1)	0.2 (0 to 0.4)	-0.3 (-0.4 to -0.2)
	Intervention 1	-0.7 (-0.8 to -0.6)		-0.3 (-0.7 to 0.2)		-0.5 (-0.7 to -0.3)	
	Intervention 1/2	-0.4 (-0.5 to -0.3)		0 (-0.3 to 0.4)		-0.2 (-0.3 to 0)	
	Intervention 1/2/3	-1.4 (-1.6 to -1.2)		-0.5 (-1.0 to 0)		-0.9 (-1.2 to -0.7)	
**≥ 65**	No policies	-0.1 (-0.2 to 0)	-0.5 (-0.6 to -0.5)	-0.2 (-0.6 to 0.2)	-0.3 (-0.6 to -0.1)	-0.2 (-0.3 to -0.1)	-0.5 (-0.5 to -0.4)
	Intervention 1	-0.9 (-1.0 to -0.8)		-0.5 (-1.1 to 0)		-0.8 (-0.9 to -0.7)	
	Intervention 1/2	-0.4 (-0.4 to -0.3)		-0.2 (-0.6 to 0.2)		-0.3 (-0.4 to -0.2)	
	Intervention 1/2/3	-0.8 (-1.0 to -0.6)		-0.5 (-1.1 to 0)		-0.7 (-0.8 to -0.5)	

CI: confidence intervals. MPC: monthly percent change. AMPC: average monthly percent change. Intervention 1: banned five highly hazardous organophosphorus (HHOP) pesticides implemented in December 2008. Intervention 2: stopped the new registration and production of paraquat parent drug and aqueous solutions and regulated the existing paraquat production and after-sales services in April 2012. Intervention 3: stopped domestic sales and use of paraquat aqueous solution in July 2016.

Appendix Table 4. Impact of pesticide policies on the standardized number of suicide by pesticide in surveillance points (MAS)

Period	Intervention 1	Intervention 2	Intervention 3	All polices
December 2008 to April 2012	2035	0	0	2035
April 2012 to July 2016	6984	-1638	0	5346
July 2016 to December 2018	5616	-1346	926	5195
Total	14634	-2984	926	12577

MAS: Mortality Attributable to Suicide. Intervention 1: banned five highly hazardous organophosphorus (HHOP) pesticides implemented in December 2008. Intervention 2: stopped the new registration and production of paraquat parent drug and aqueous solutions and regulated the existing paraquat production and after-sales services in April 2012. Intervention 3: stopped domestic sales and use of paraquat aqueous solution in July 2016.

Appendix Figure 1. The pesticide suicide trend of changes after the intervention effect lagged in China

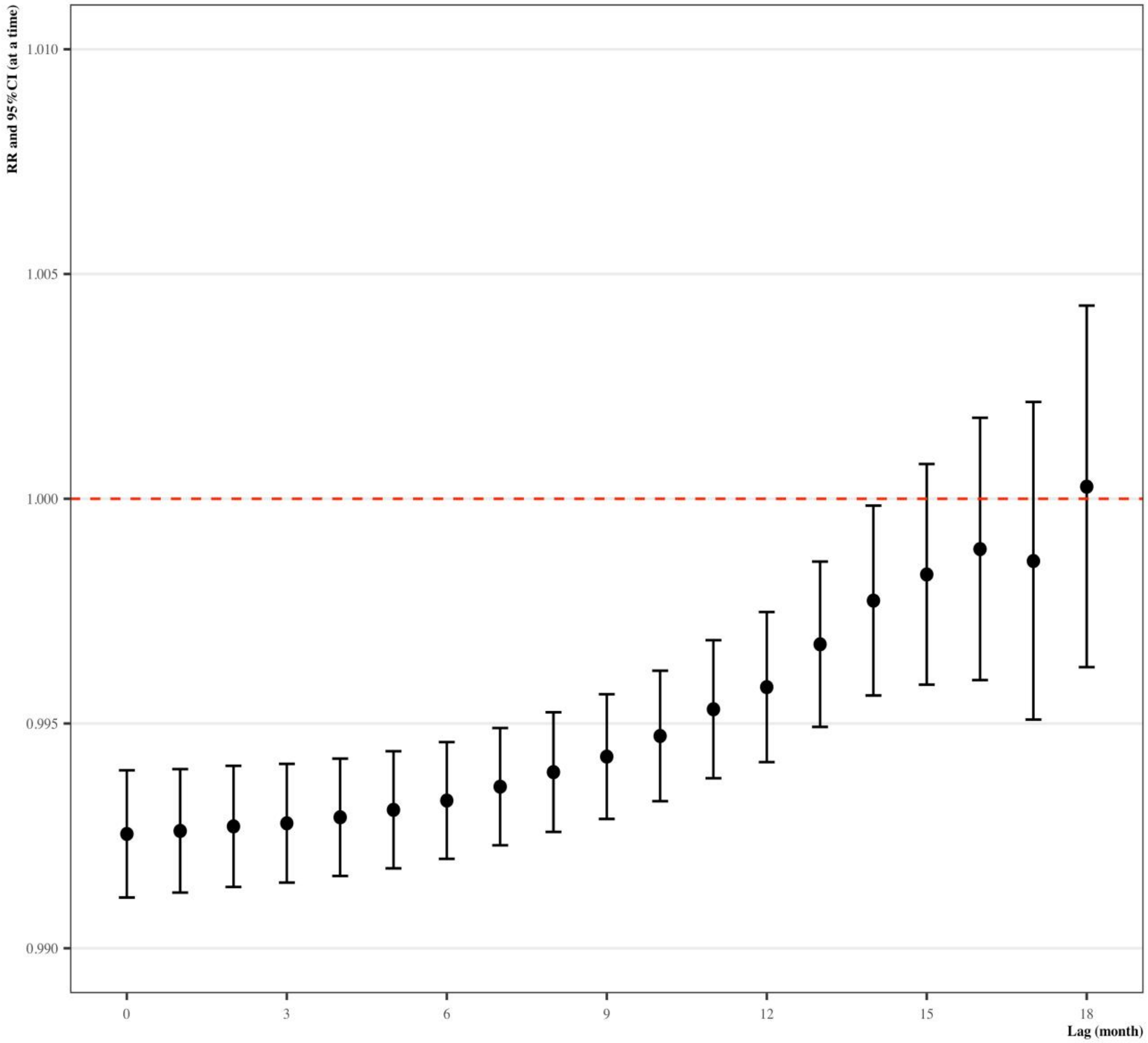

Intervention 1: banned five highly hazardous organophosphorus (HHOP) pesticides implemented in December 2008.

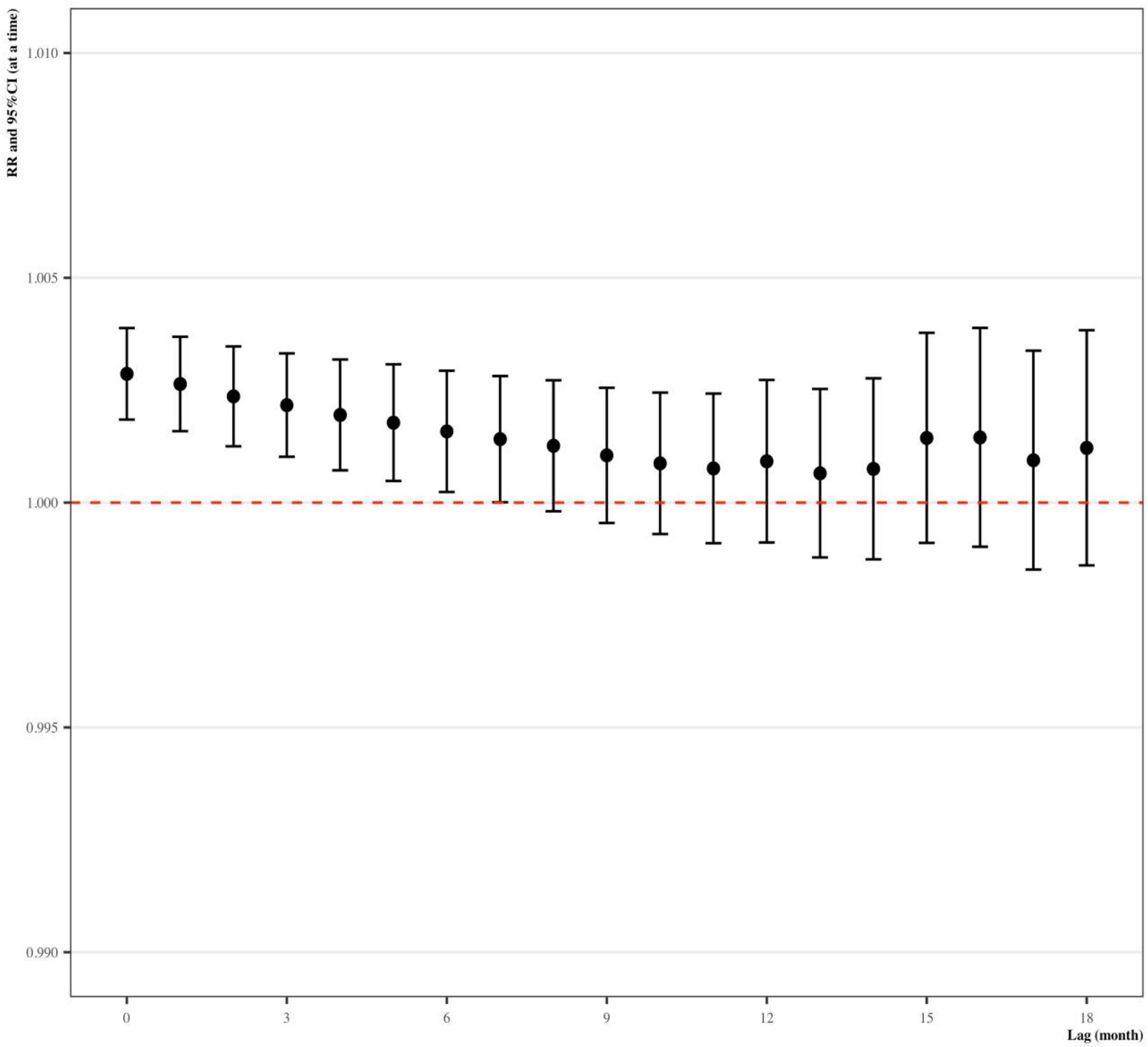

Intervention 2: stopped the new registration and production of paraquat parent drug and aqueous solutions and regulated the existing paraquat production and after-sales services in April 2012.

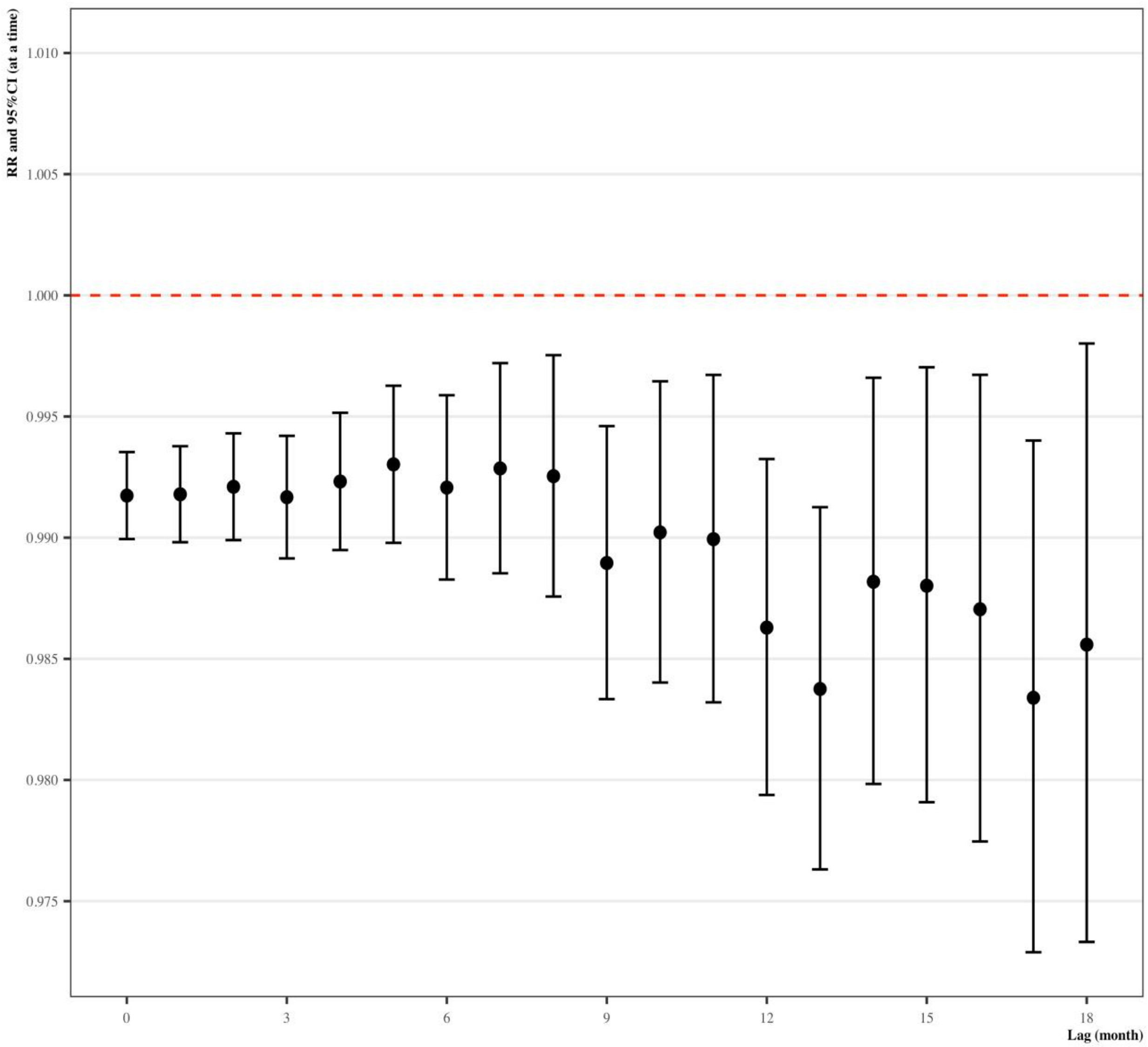

Intervention 3: stopped domestic sales and use of paraquat aqueous solution in July 2016.

Appendix Figure 2. The trend of monthly suicide, pesticide suicide and non-pesticide suicide rate and corresponding counterfactual in China from Jan 2006 to Dec 2018

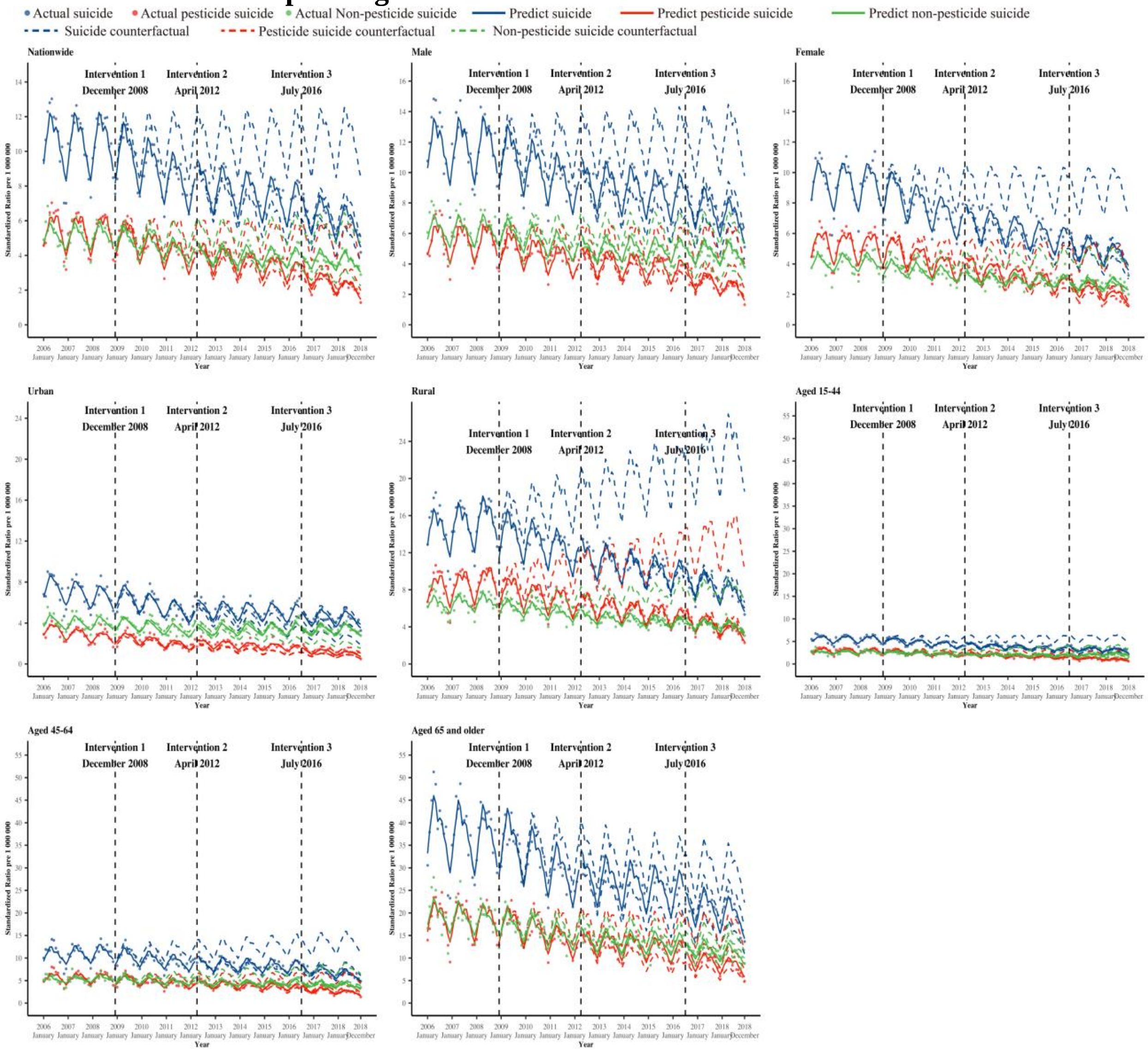

Intervention 1: banned five highly hazardous organophosphorus (HHOP) pesticides implemented in December 2008. Intervention 2: stopped the new registration and production of paraquat parent drug and aqueous solutions and regulated the existing paraquat production and after-sales services in April 2012. Intervention 3: stopped domestic sales and use of paraquat aqueous solution in July 2016.

Appendix Figure 3. The model residual autocorrelation and partial autocorrelation test before and after the model adjusted

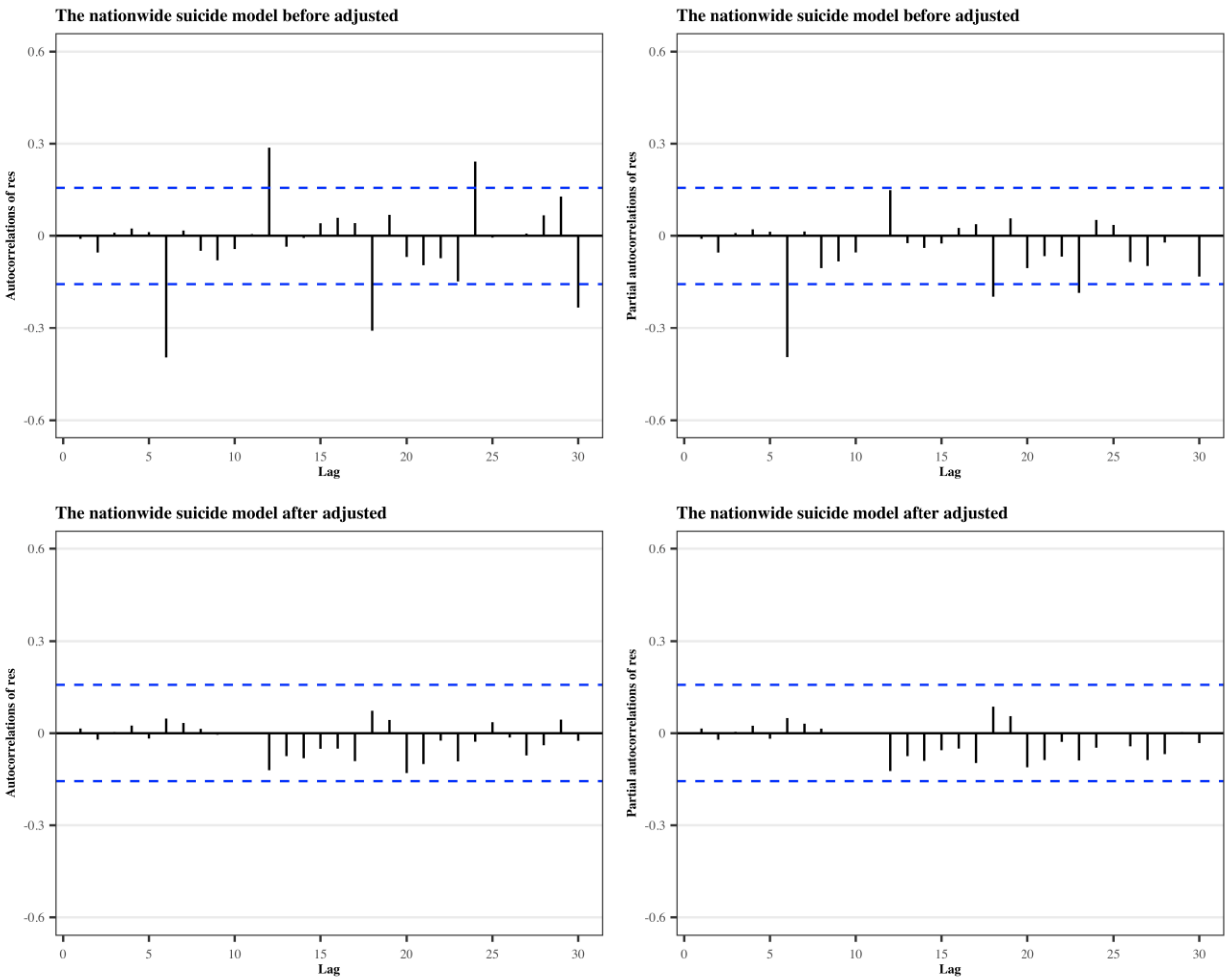
The nationwide suicide model before adjusted
Autocorrelations of res
0.6
0.3
0
-0.3
-0.6
0
5
10
15
20
25
30
Lag
The nationwide suicide model before adjusted
Partial autocorrelations of res
0.6
0.3
0
-0.3
-0.6
0
5
10
15
20
25
30
Lag
The nationwide suicide model after adjusted
Autocorrelations of res
0.6
0.3
0
-0.3
-0.6
0
5
10
15
20
25
30
Lag
The nationwide suicide model after adjusted
Partial autocorrelations of res
0.6
0.3
0
-0.3
-0.6
0
5
10
15
20
25
30
Lag

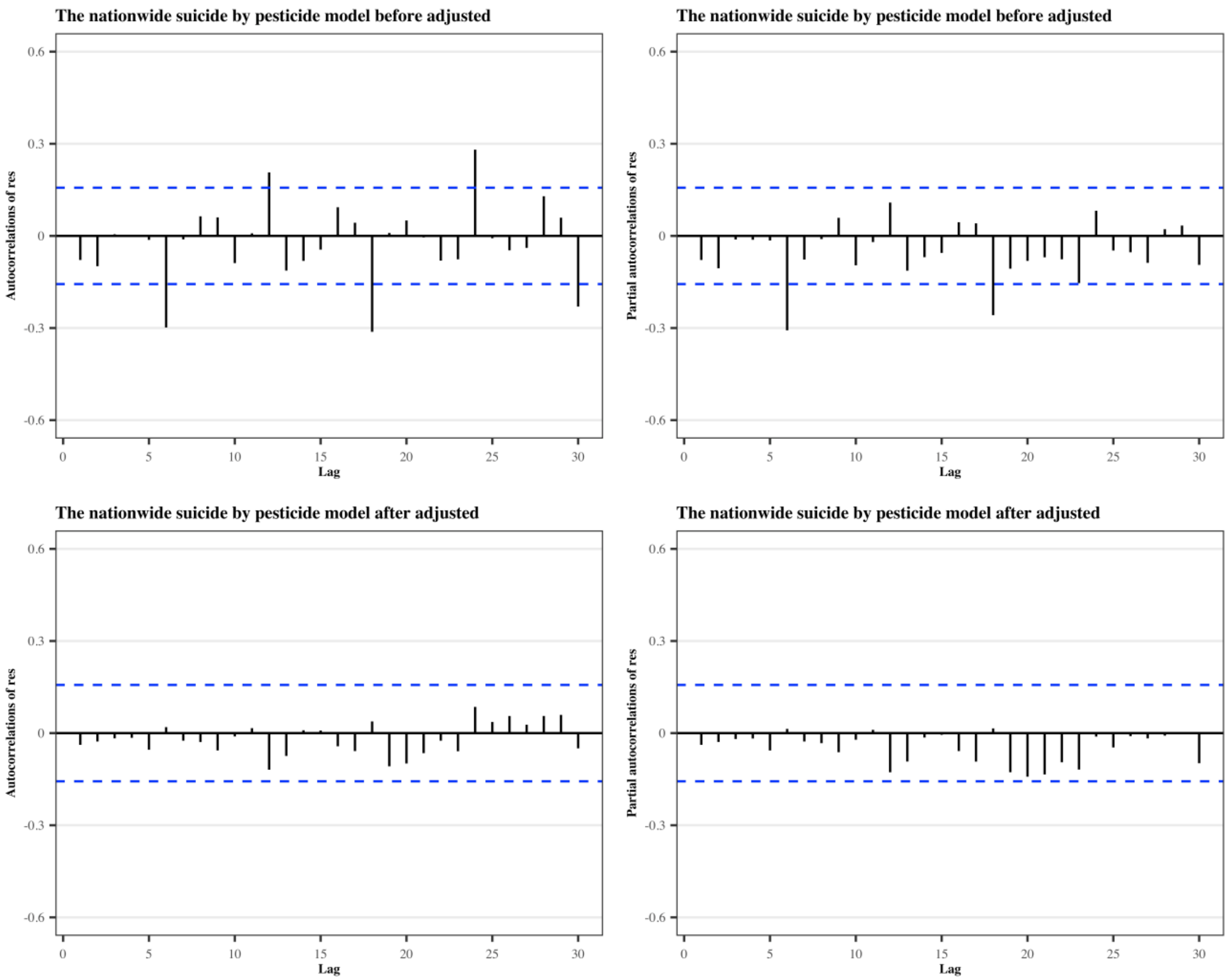
The nationwide suicide by pesticide model before adjusted
Autocorrelations of res
Partial autocorrelations of res
The nationwide suicide by pesticide model after adjusted
Lag
0.6
0.3
0
-0.3
-0.6
0
5
10
15
20
25
30

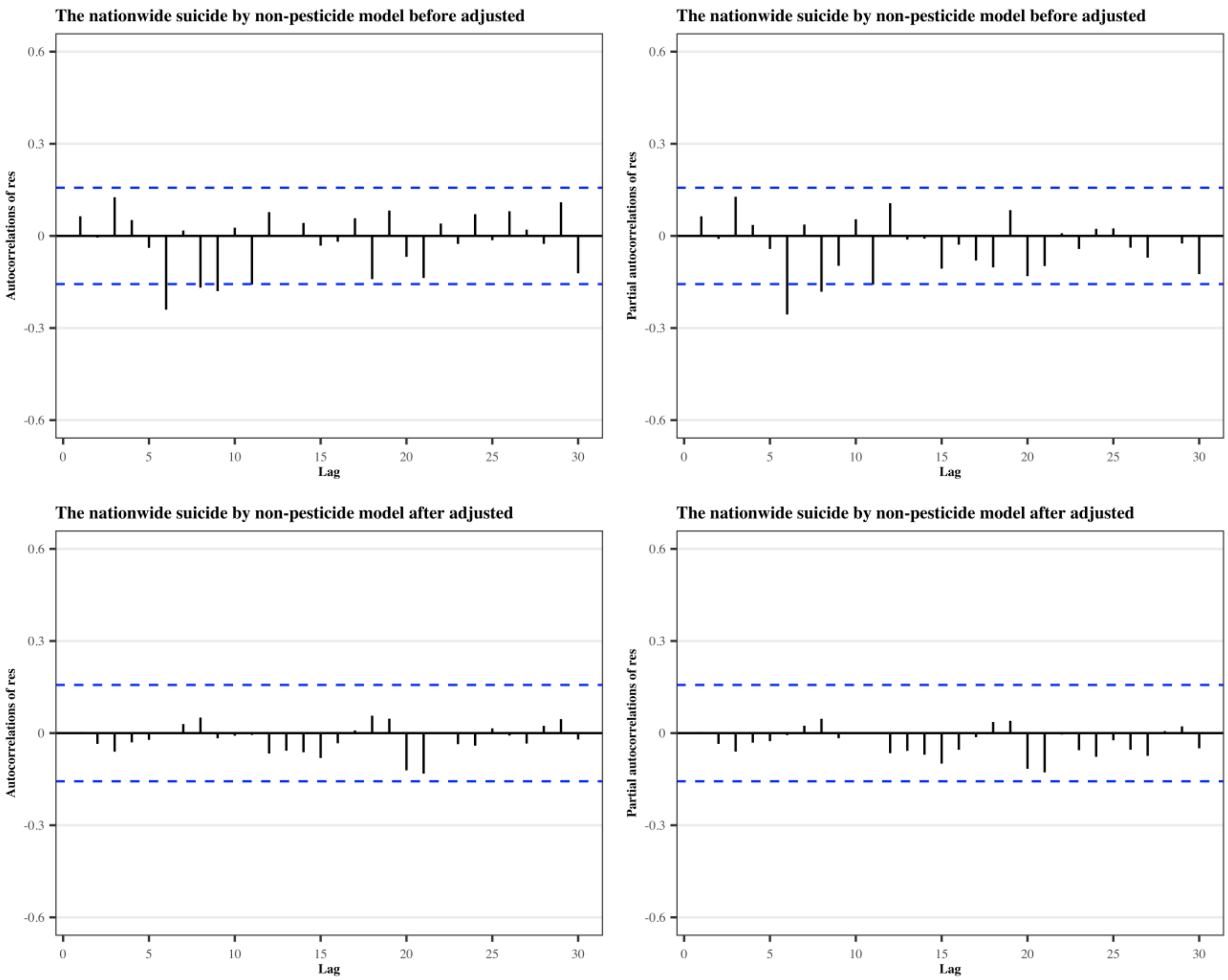

The nationwide suicide by non-pesticide model before adjusted
Autocorrelations of res
Partial autocorrelations of res
The nationwide suicide by non-pesticide model after adjusted
Lag
0.6
0.3
0
-0.3
-0.6
0
5
10
15
20
25
30

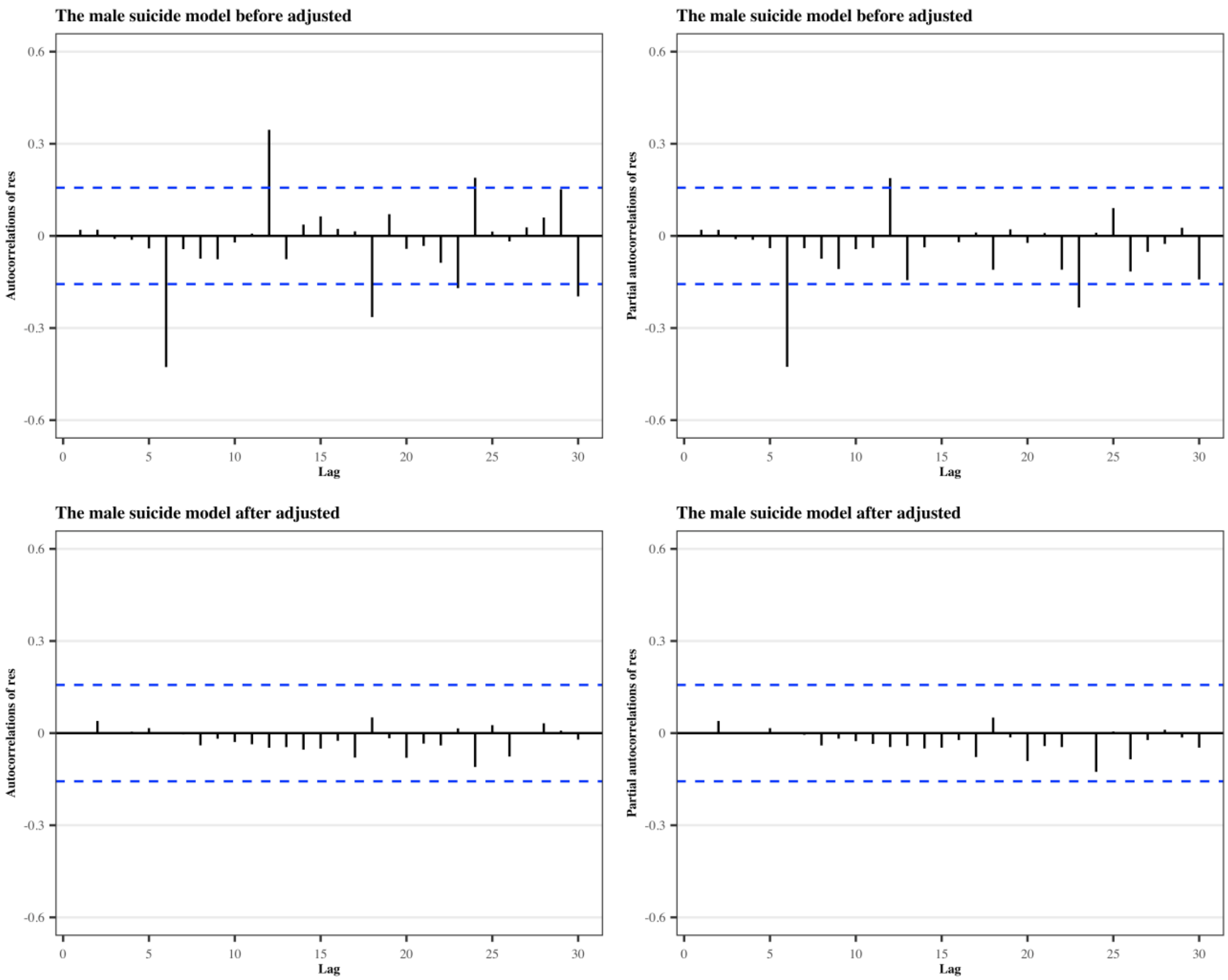

The male suicide model before adjusted
Autocorrelations of res
0.6
0.3
0
-0.3
-0.6
0
5
10
15
20
25
30
Lag
The male suicide model before adjusted
Partial autocorrelations of res
Lag
The male suicide model after adjusted
Autocorrelations of res
Lag
The male suicide model after adjusted
Partial autocorrelations of res
Lag

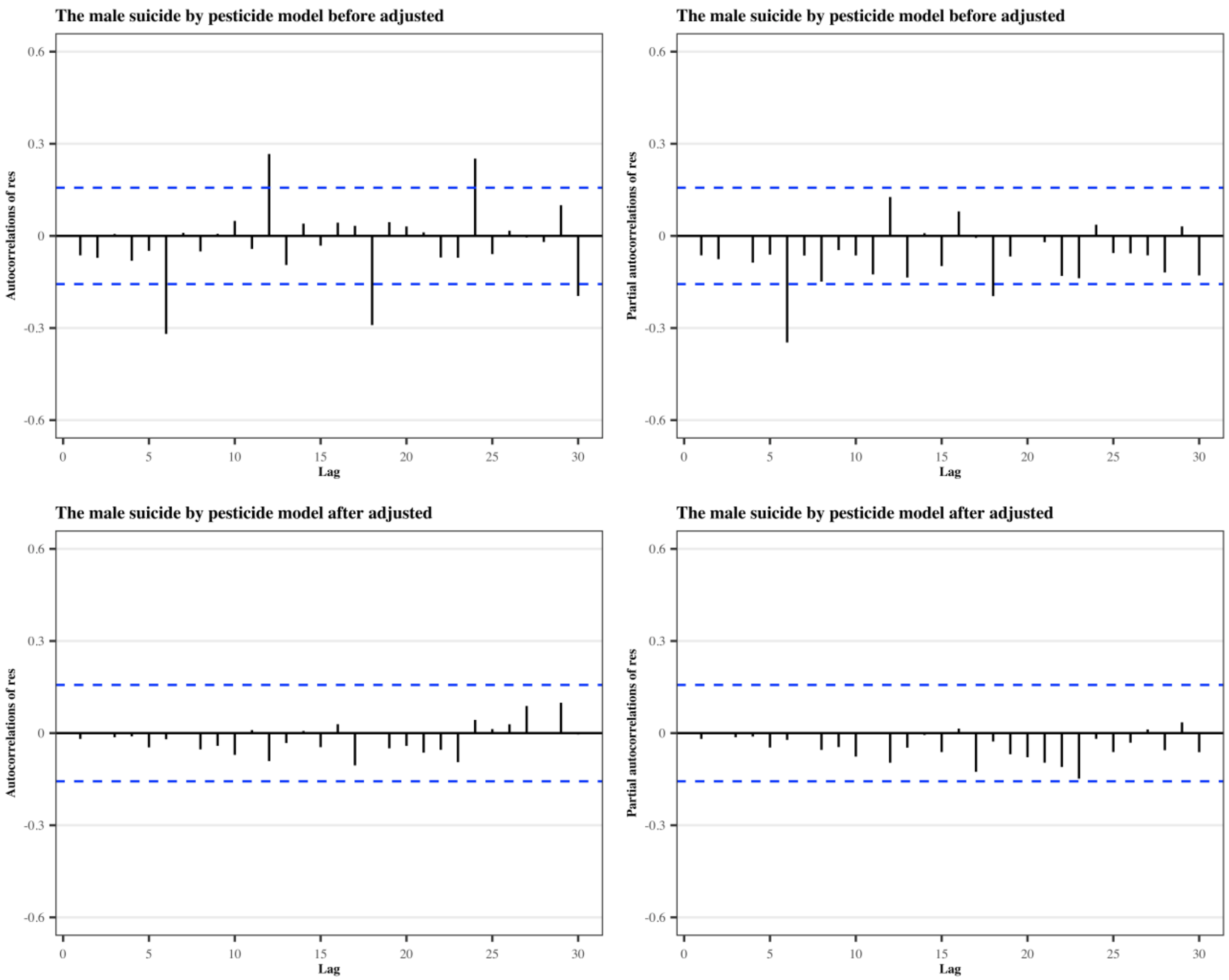
The male suicide by pesticide model before adjusted
Autocorrelations of res
Lag
The male suicide by pesticide model before adjusted
Partial autocorrelations of res
Lag
The male suicide by pesticide model after adjusted
Autocorrelations of res
Lag
The male suicide by pesticide model after adjusted
Partial autocorrelations of res
Lag
0.6
0.3
0
-0.3
-0.6
0
5
10
15
20
25
30

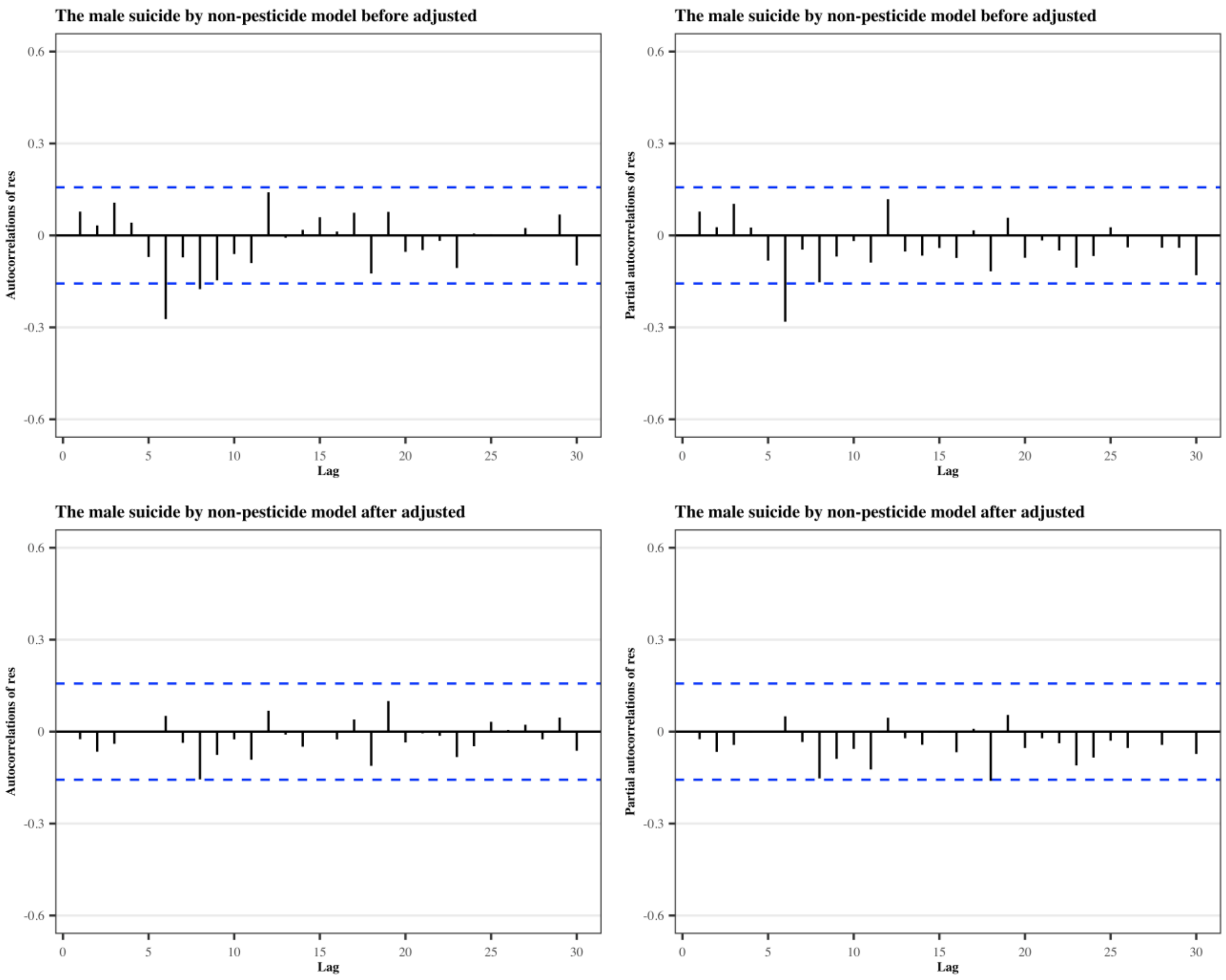
The male suicide by non-pesticide model before adjusted
Autocorrelations of res
Lag
The male suicide by non-pesticide model before adjusted
Partial autocorrelations of res
Lag
The male suicide by non-pesticide model after adjusted
Autocorrelations of res
Lag
The male suicide by non-pesticide model after adjusted
Partial autocorrelations of res
Lag

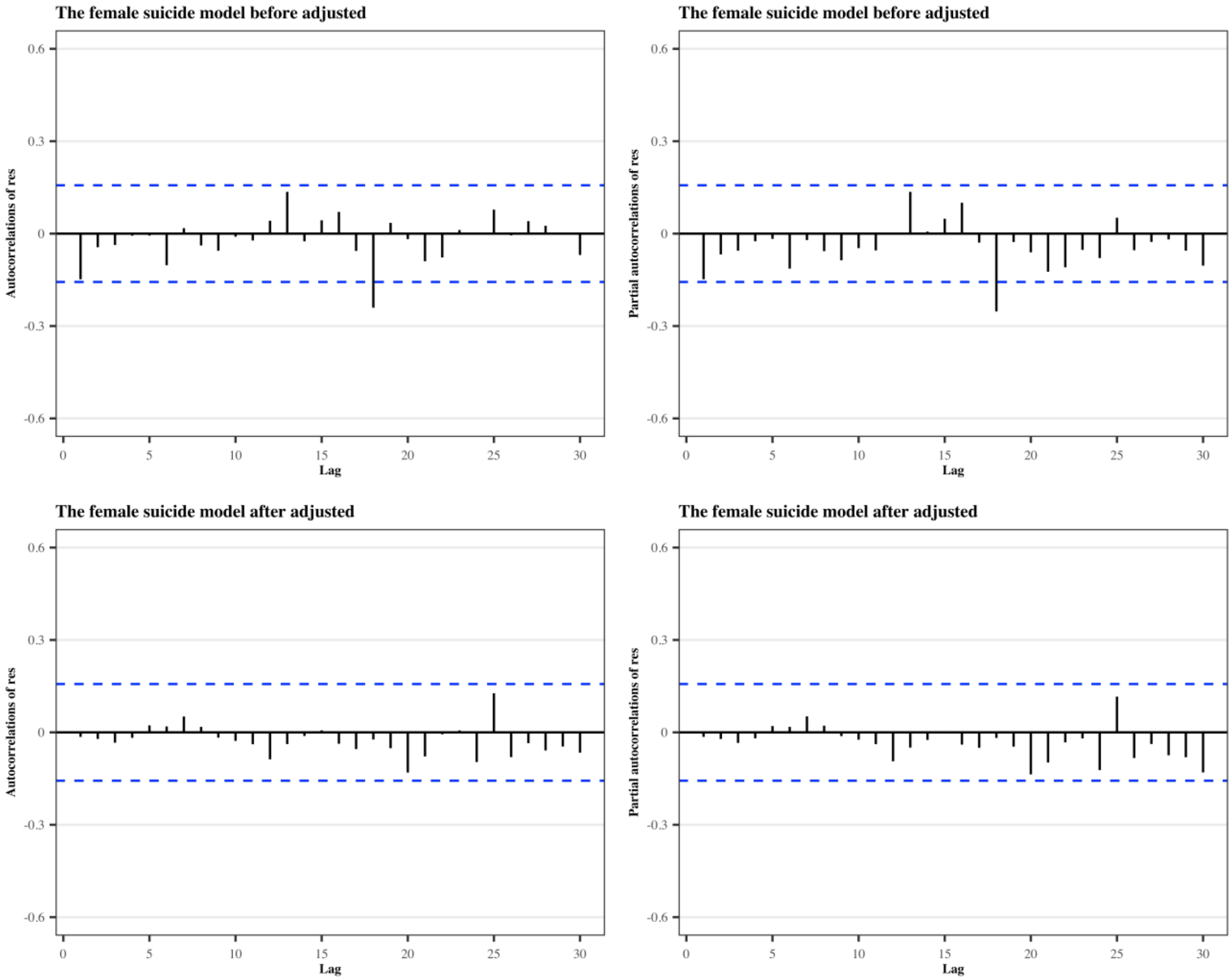
The female suicide model before adjusted
Autocorrelations of res
Lag
The female suicide model before adjusted
Partial autocorrelations of res
Lag
The female suicide model after adjusted
Autocorrelations of res
Lag
The female suicide model after adjusted
Partial autocorrelations of res
Lag
0.6
0.3
0
-0.3
-0.6
0
5
10
15
20
25
30

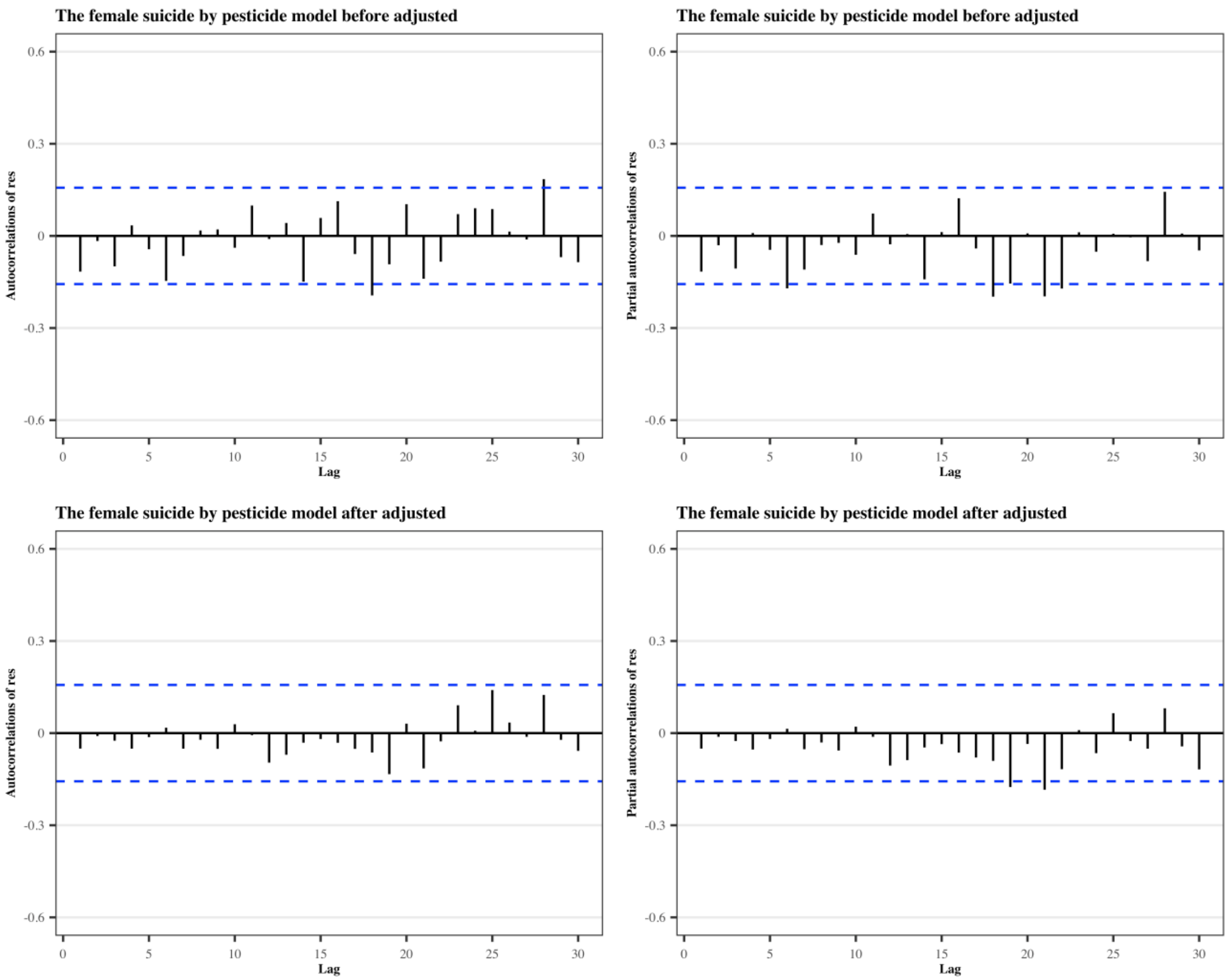
The female suicide by pesticide model before adjusted
Autocorrelations of res
Lag
The female suicide by pesticide model before adjusted
Partial autocorrelations of res
Lag
The female suicide by pesticide model after adjusted
Autocorrelations of res
Lag
The female suicide by pesticide model after adjusted
Partial autocorrelations of res
Lag
0.6
0.3
0
-0.3
-0.6
0
5
10
15
20
25
30

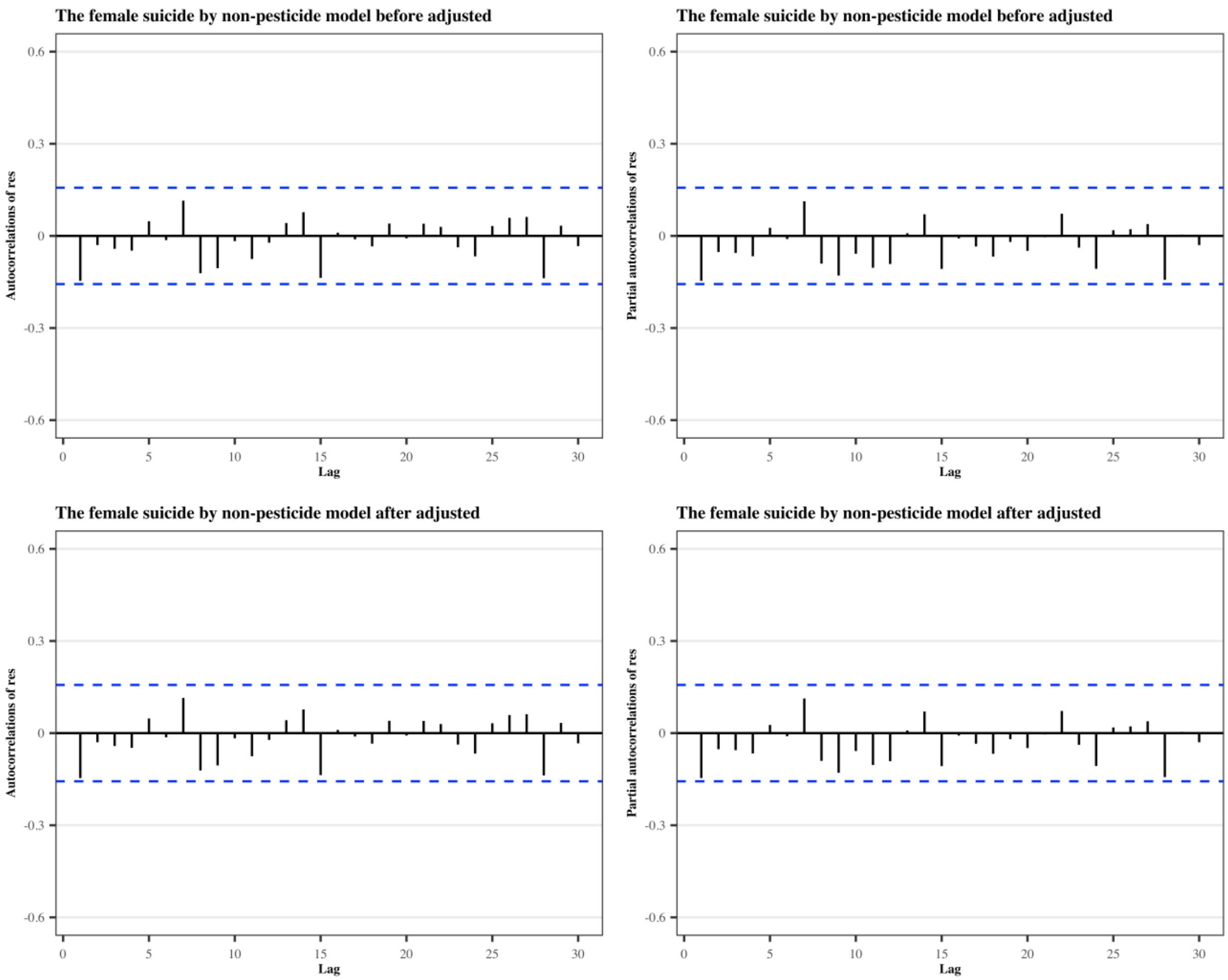
The female suicide by non-pesticide model before adjusted
Autocorrelations of res
Lag
The female suicide by non-pesticide model before adjusted
Partial autocorrelations of res
Lag
The female suicide by non-pesticide model after adjusted
Autocorrelations of res
Lag
The female suicide by non-pesticide model after adjusted
Partial autocorrelations of res
Lag
0.6
0.3
0
-0.3
-0.6
0
5
10
15
20
25
30

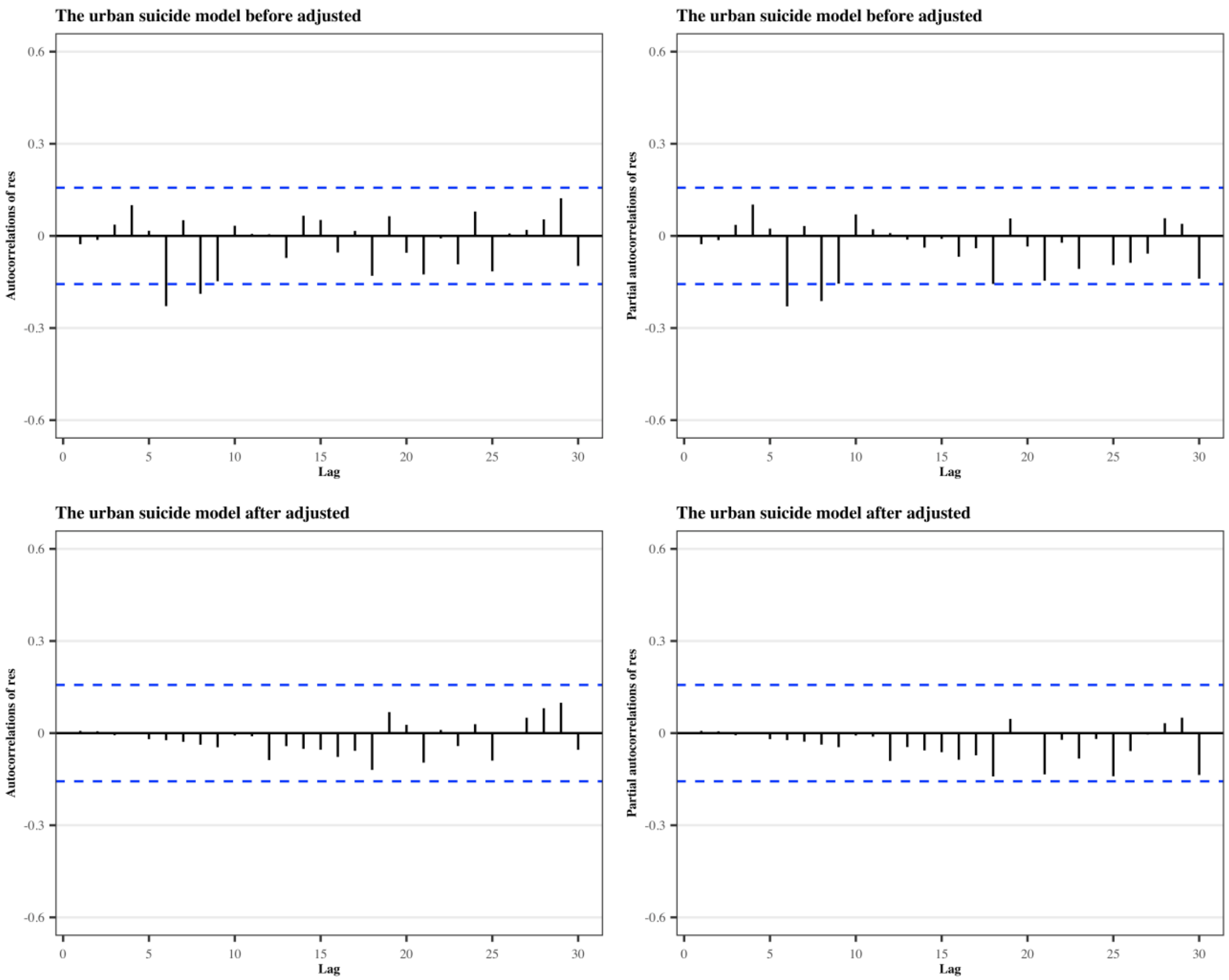
The urban suicide model before adjusted
Autocorrelations of res
Lag
The urban suicide model before adjusted
Partial autocorrelations of res
Lag
The urban suicide model after adjusted
Autocorrelations of res
Lag
The urban suicide model after adjusted
Partial autocorrelations of res
Lag
0.6
0.3
0
-0.3
-0.6
0
5
10
15
20
25
30

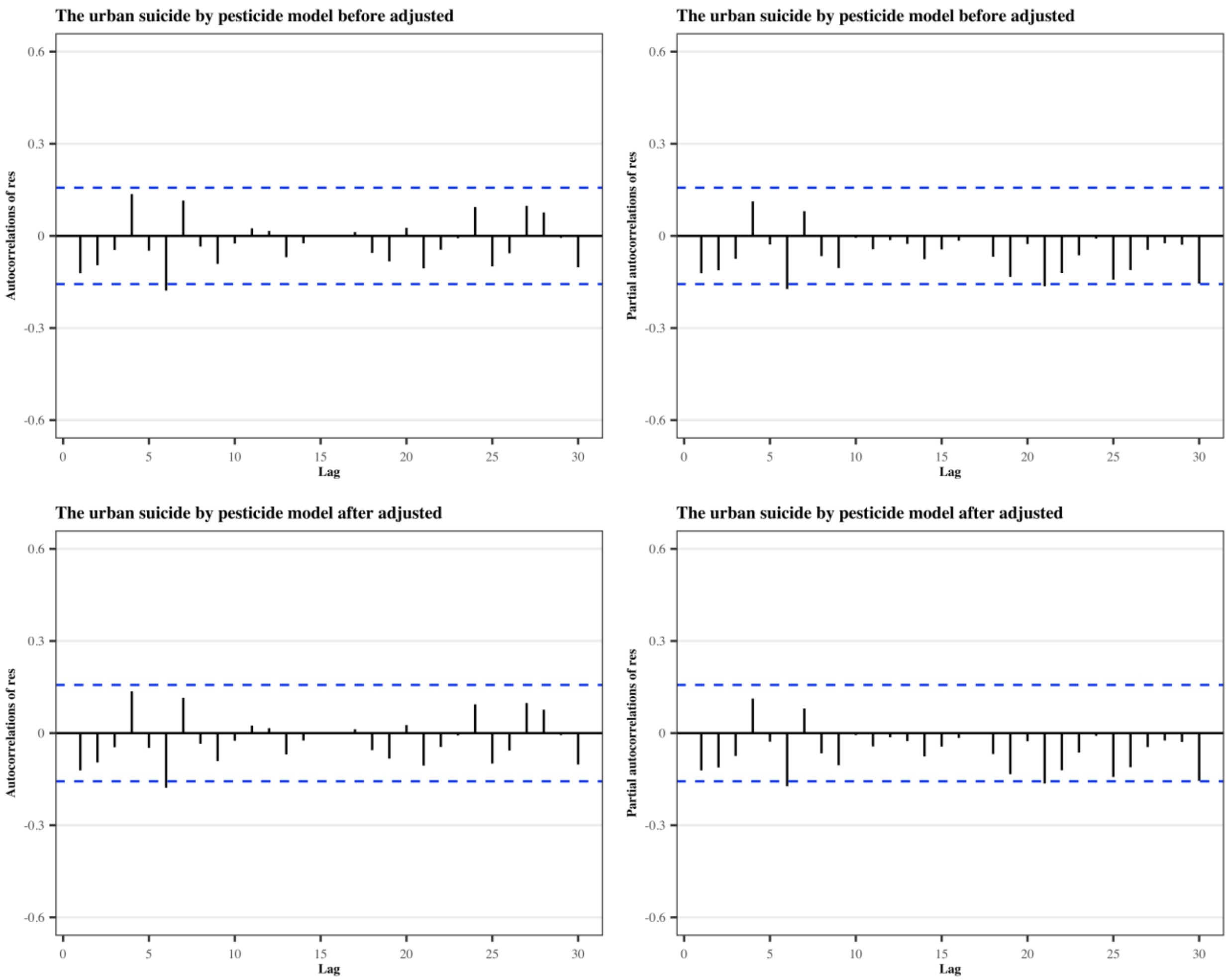
The urban suicide by pesticide model before adjusted
Autocorrelations of res
The urban suicide by pesticide model before adjusted
Partial autocorrelations of res
The urban suicide by pesticide model after adjusted
Autocorrelations of res
The urban suicide by pesticide model after adjusted
Partial autocorrelations of res
0.6
0.3
0
-0.3
-0.6
0
5
10
15
20
25
30
Lag

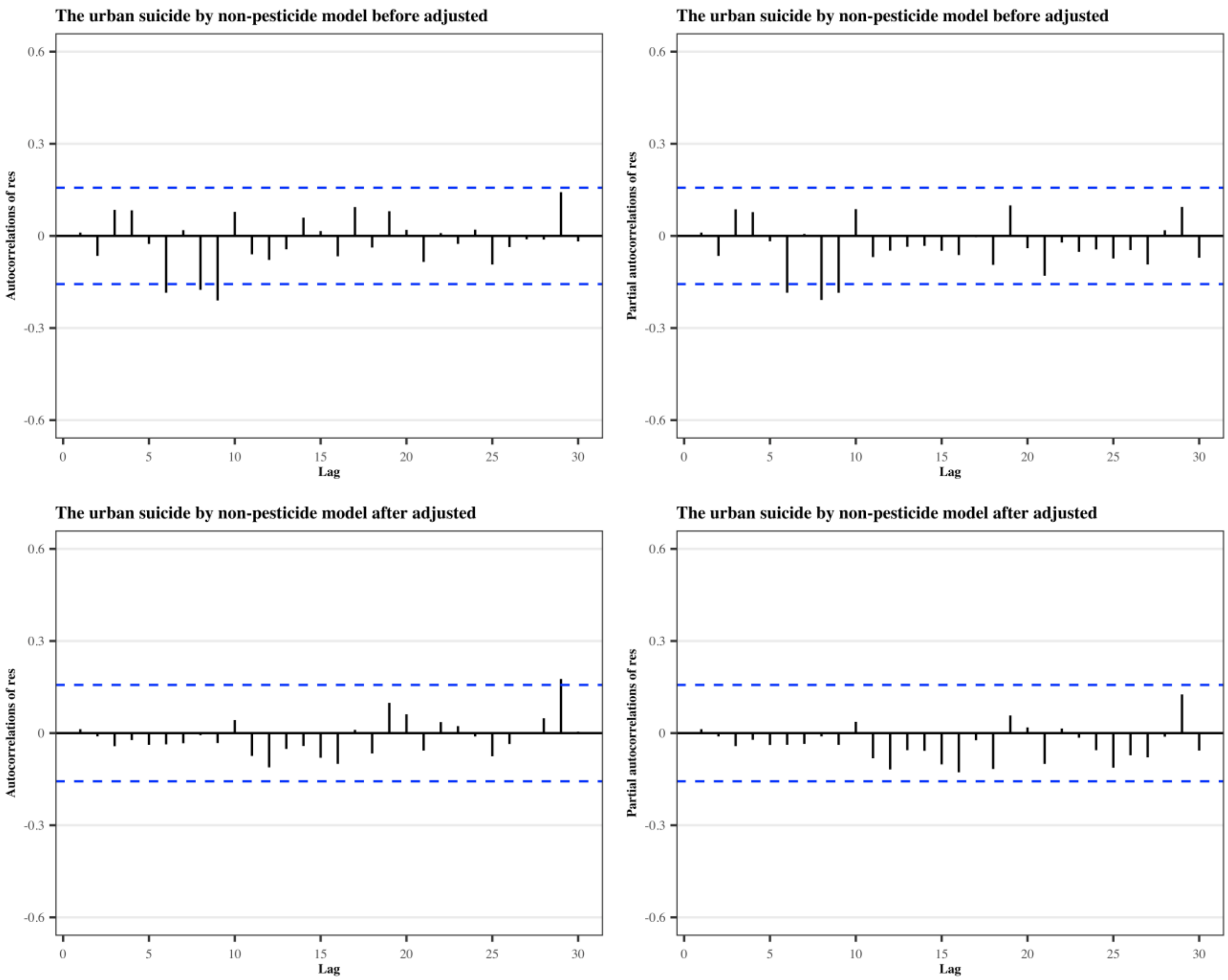
The urban suicide by non-pesticide model before adjusted
Autocorrelations of res
Lag
The urban suicide by non-pesticide model before adjusted
Partial autocorrelations of res
Lag
The urban suicide by non-pesticide model after adjusted
Autocorrelations of res
Lag
The urban suicide by non-pesticide model after adjusted
Partial autocorrelations of res
Lag
0.6
0.3
0
-0.3
-0.6
0
5
10
15
20
25
30

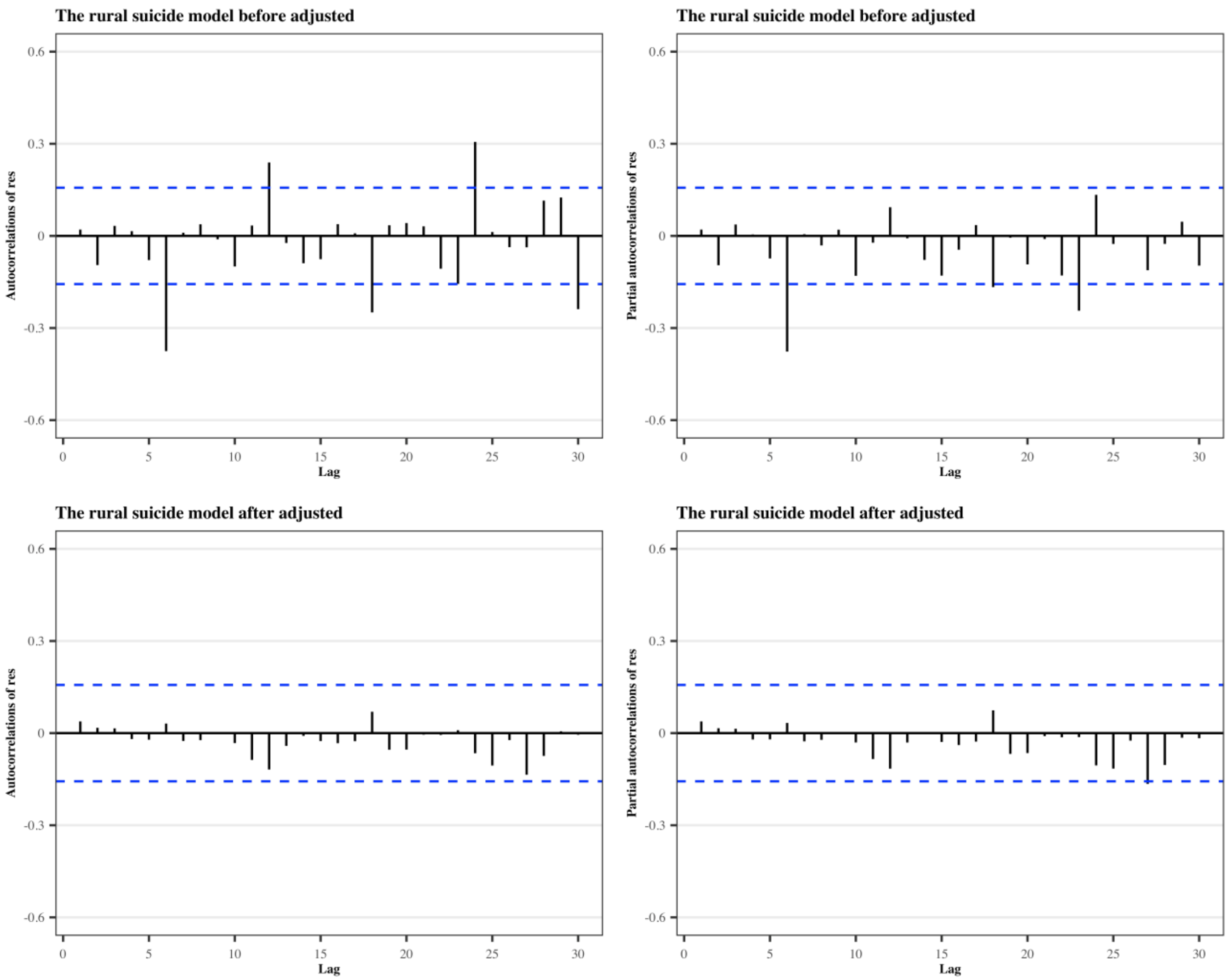
The rural suicide model before adjusted
Autocorrelations of res
Lag
The rural suicide model before adjusted
Partial autocorrelations of res
Lag
The rural suicide model after adjusted
Autocorrelations of res
Lag
The rural suicide model after adjusted
Partial autocorrelations of res
Lag

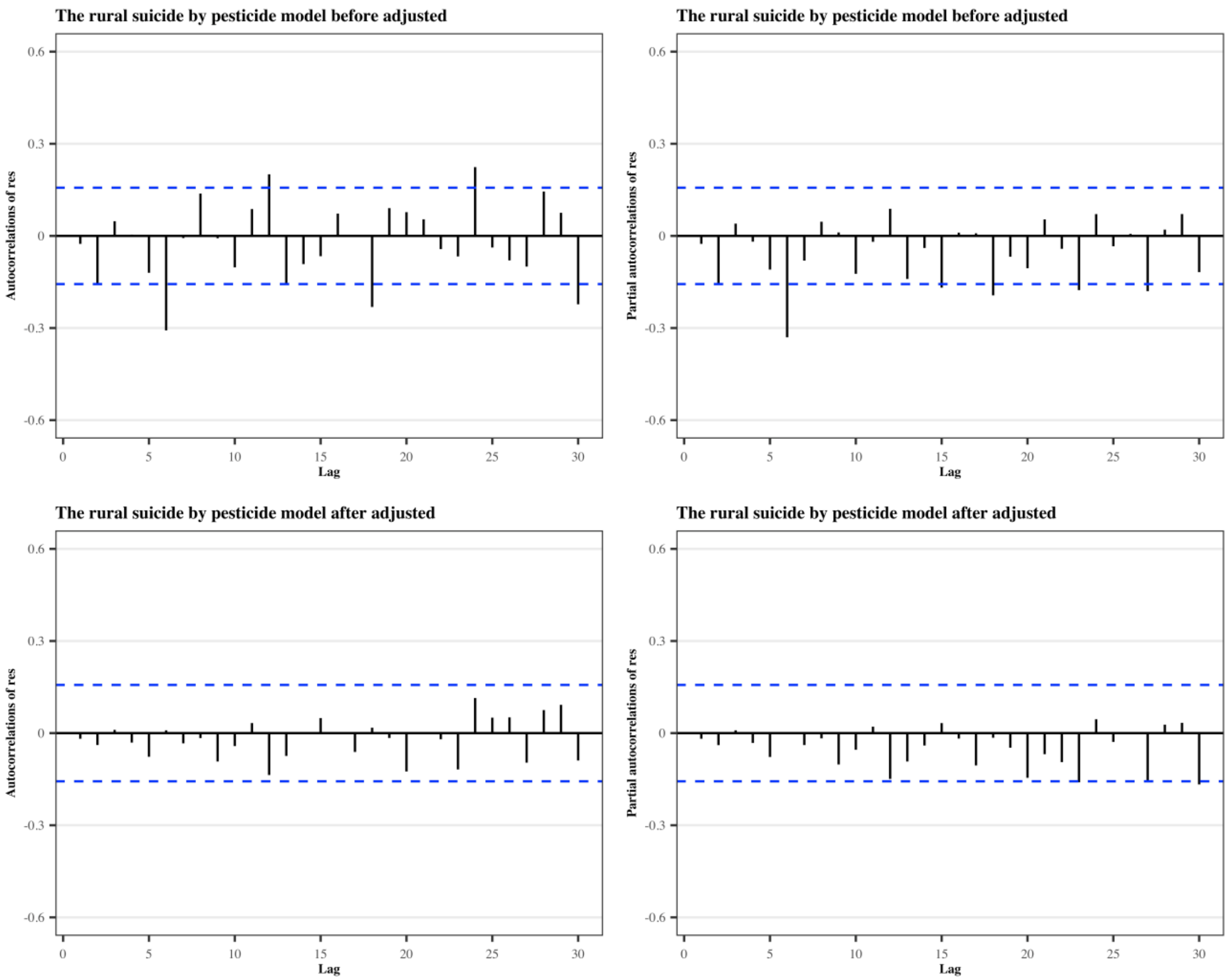
The rural suicide by pesticide model before adjusted
Autocorrelations of res
0.6
0.3
0
-0.3
-0.6
0
5
10
15
20
25
30
Lag
The rural suicide by pesticide model before adjusted
Partial autocorrelations of res
Lag
The rural suicide by pesticide model after adjusted
Autocorrelations of res
Lag
The rural suicide by pesticide model after adjusted
Partial autocorrelations of res
Lag

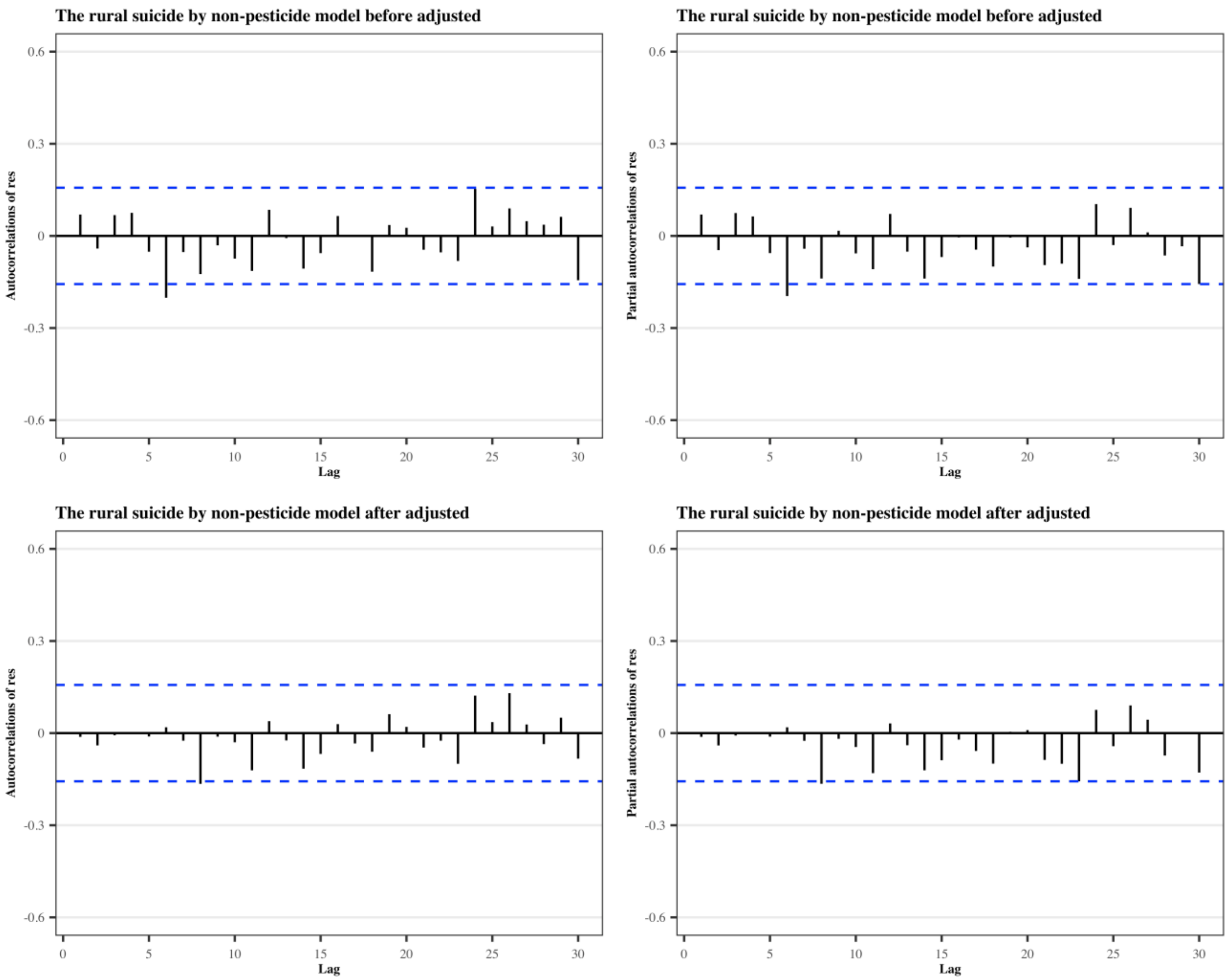
The rural suicide by non-pesticide model before adjusted
Autocorrelations of res
Partial autocorrelations of res
The rural suicide by non-pesticide model after adjusted
Lag
0.6
0.3
0
-0.3
-0.6
0
5
10
15
20
25
30

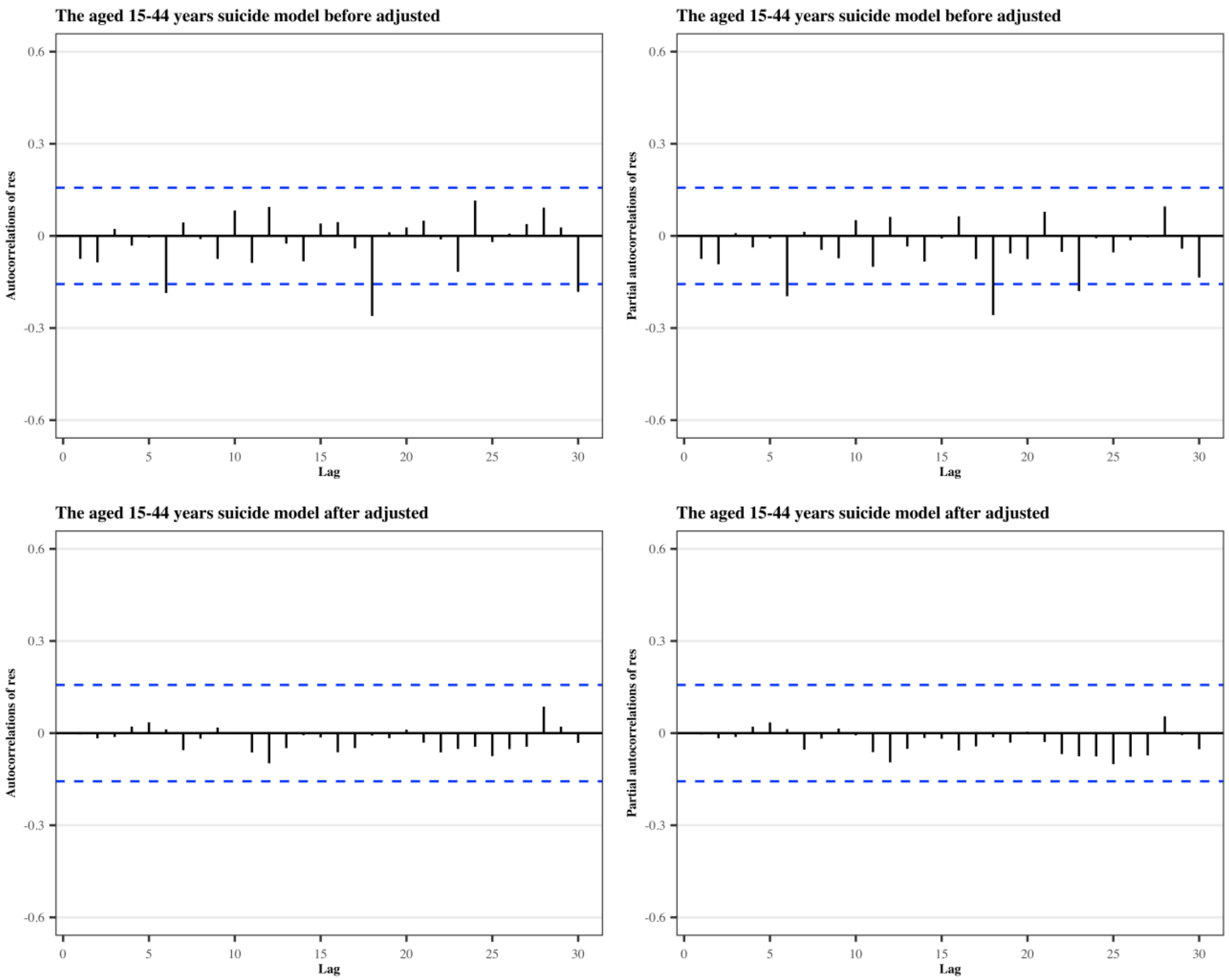
The aged 15-44 years suicide model before adjusted
The aged 15-44 years suicide model before adjusted
The aged 15-44 years suicide model after adjusted
The aged 15-44 years suicide model after adjusted
Autocorrelations of res
Partial autocorrelations of res
Lag
0.6
0.3
0
-0.3
-0.6
0
5
10
15
20
25
30

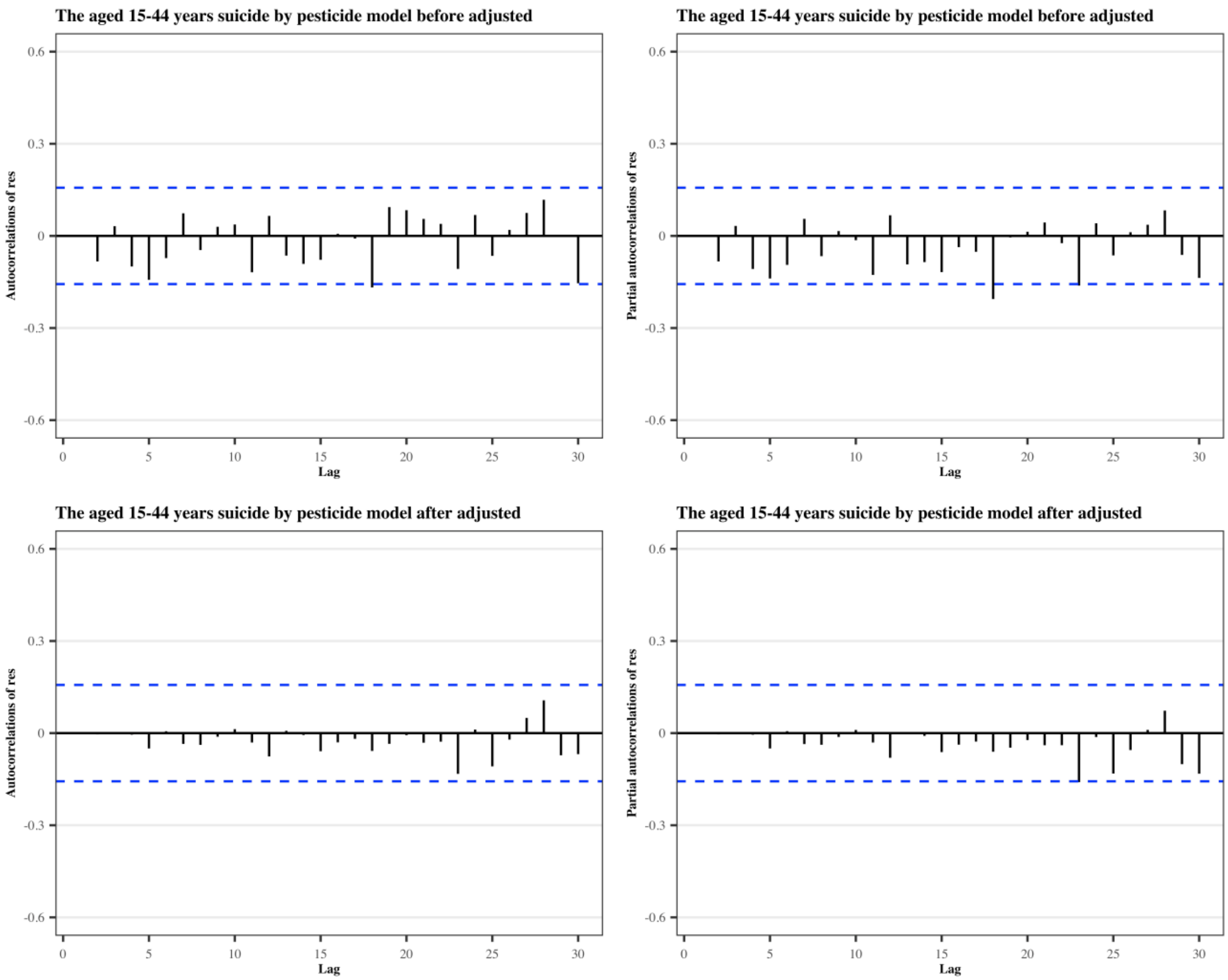

The aged 15-44 years suicide by pesticide model before adjusted
Autocorrelations of res
Lag
The aged 15-44 years suicide by pesticide model before adjusted
Partial autocorrelations of res
Lag
The aged 15-44 years suicide by pesticide model after adjusted
Autocorrelations of res
Lag
The aged 15-44 years suicide by pesticide model after adjusted
Partial autocorrelations of res
Lag

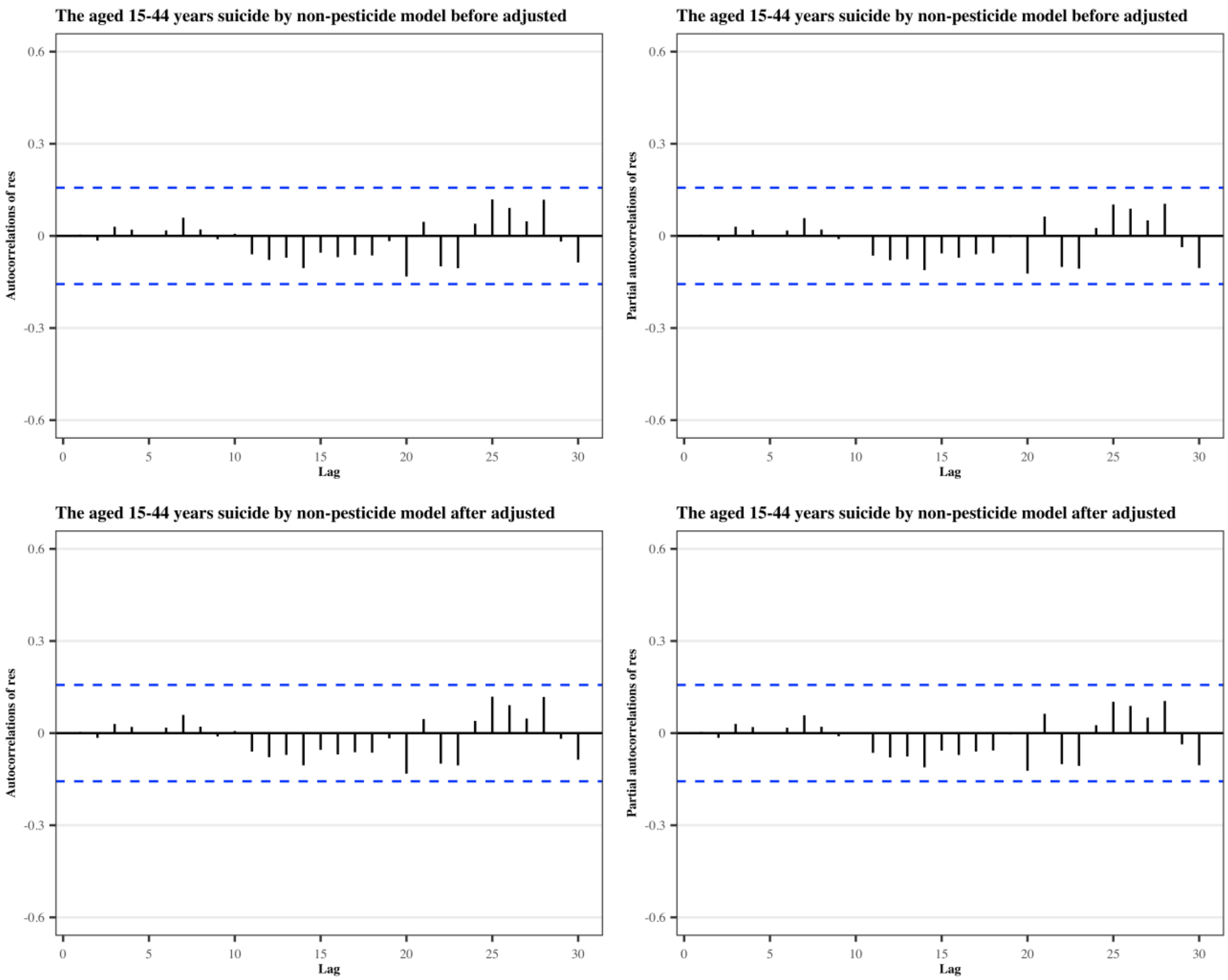
The aged 15-44 years suicide by non-pesticide model before adjusted
Autocorrelations of res
Partial autocorrelations of res
The aged 15-44 years suicide by non-pesticide model after adjusted
Lag
0.6
0.3
0
-0.3
-0.6
0
5
10
15
20
25
30

The aged 45-64 years suicide model before adjusted

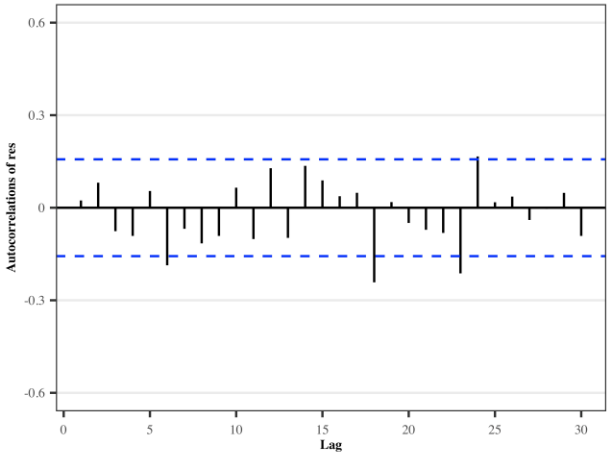

The aged 45-64 years suicide model before adjusted

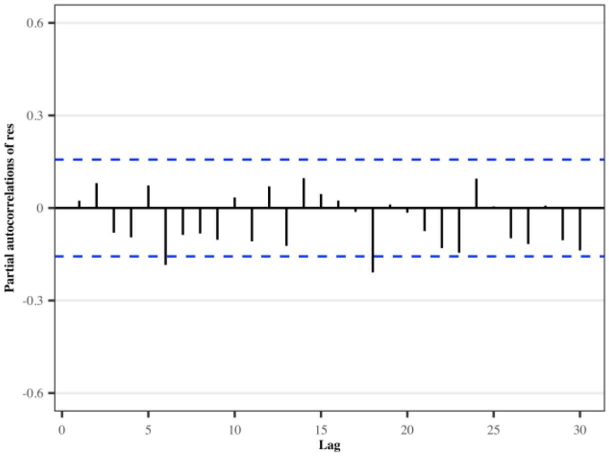

The aged 45-64 years suicide model after adjusted

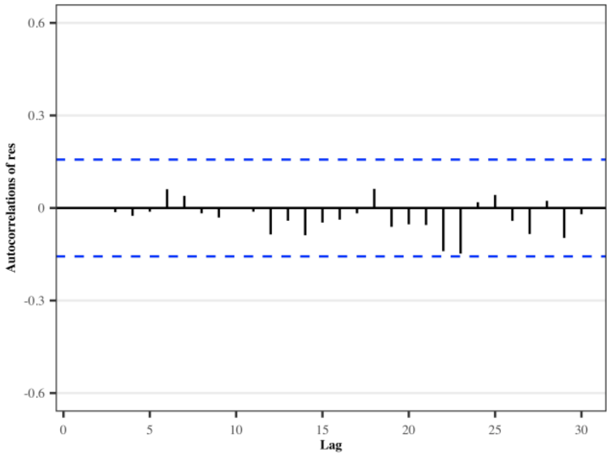

The aged 45-64 years suicide model after adjusted

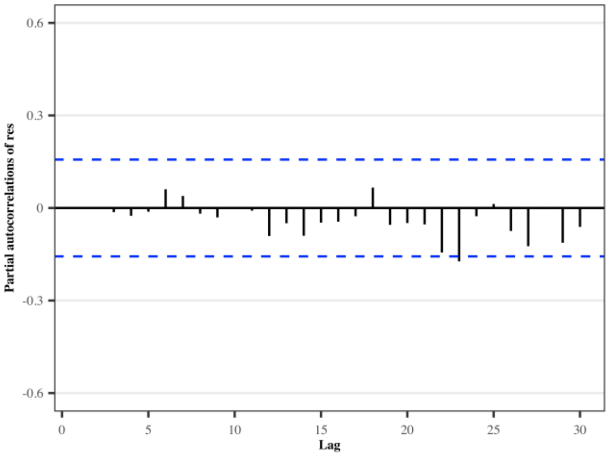

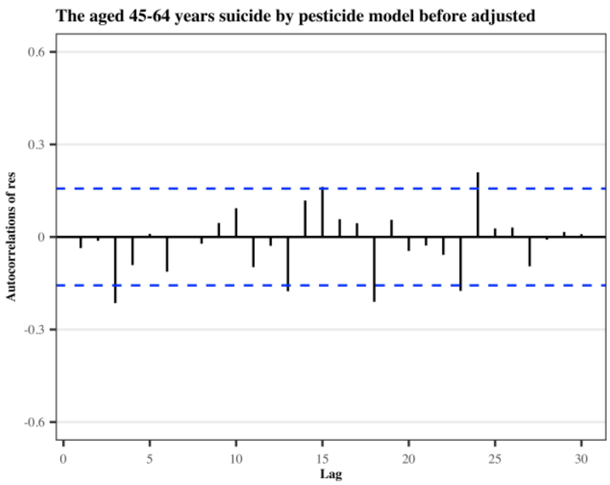
The aged 45-64 years suicide by pesticide model before adjusted
Autocorrelations of res
Lag

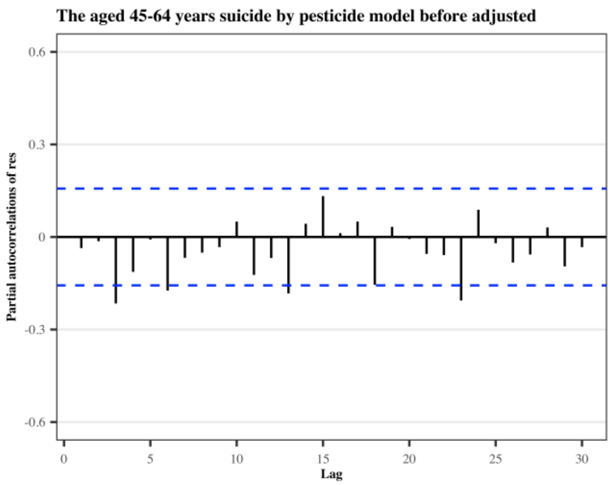
The aged 45-64 years suicide by pesticide model before adjusted
Partial autocorrelations of res
Lag

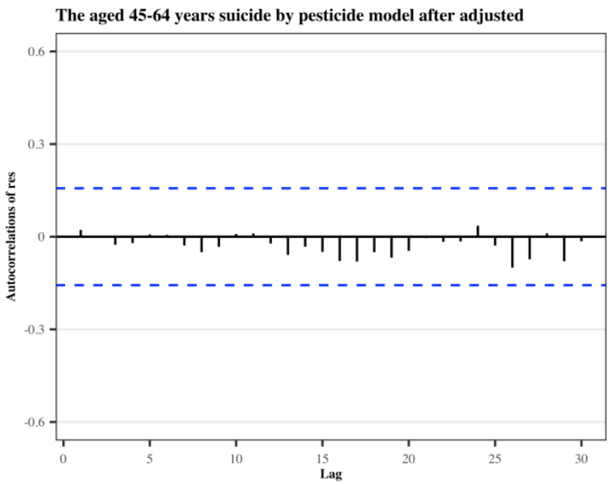
The aged 45-64 years suicide by pesticide model after adjusted
Autocorrelations of res
Lag

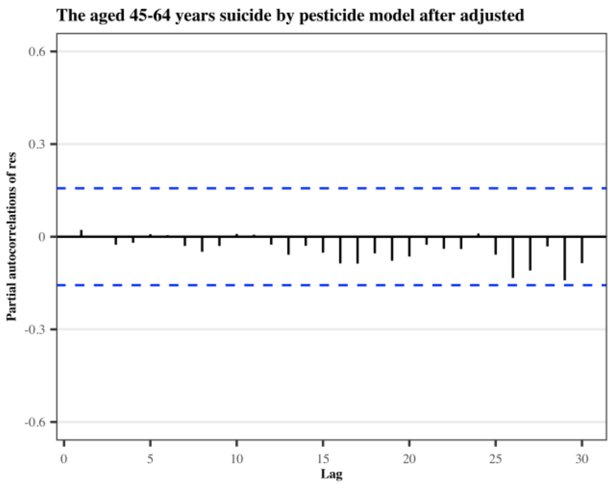
The aged 45-64 years suicide by pesticide model after adjusted
Partial autocorrelations of res
Lag

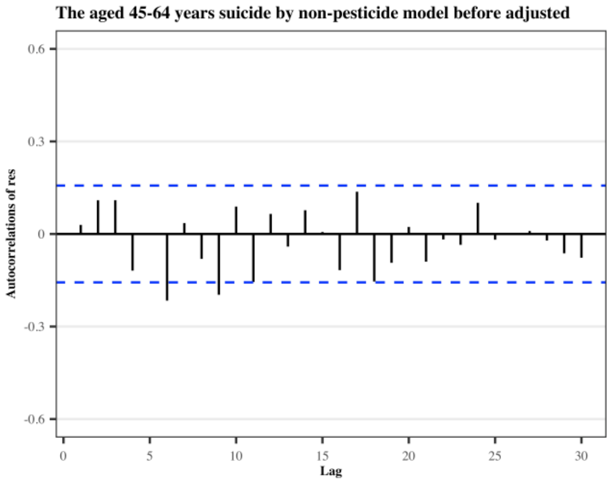
The aged 45-64 years suicide by non-pesticide model before adjusted
Autocorrelations of res
Lag
0.6
0.3
0
-0.3
-0.6
0
5
10
15
20
25
30

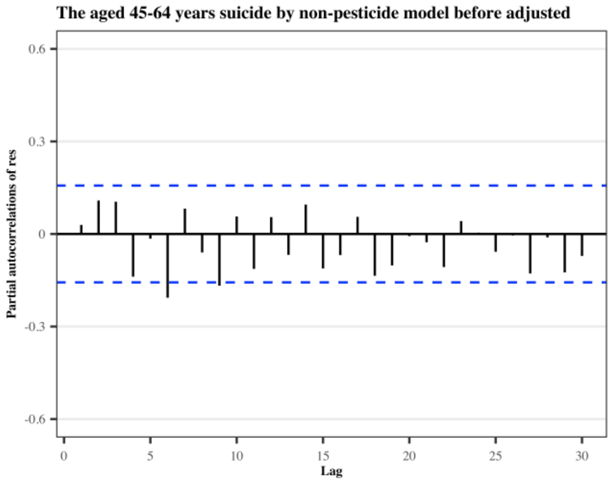
The aged 45-64 years suicide by non-pesticide model before adjusted
Partial autocorrelations of res
Lag
0.6
0.3
0
-0.3
-0.6
0
5
10
15
20
25
30

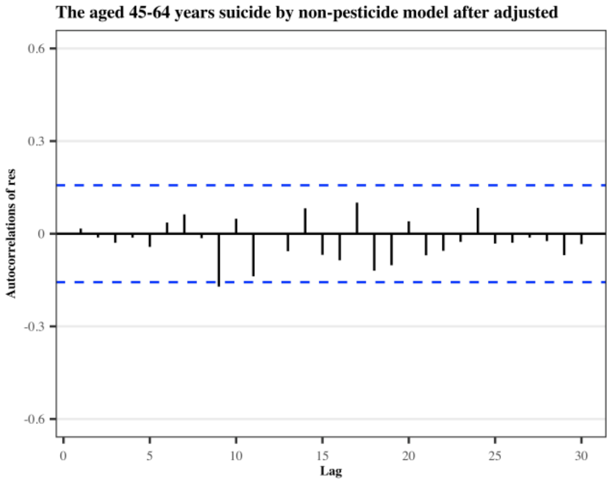
The aged 45-64 years suicide by non-pesticide model after adjusted
Autocorrelations of res
Lag
0.6
0.3
0
-0.3
-0.6
0
5
10
15
20
25
30

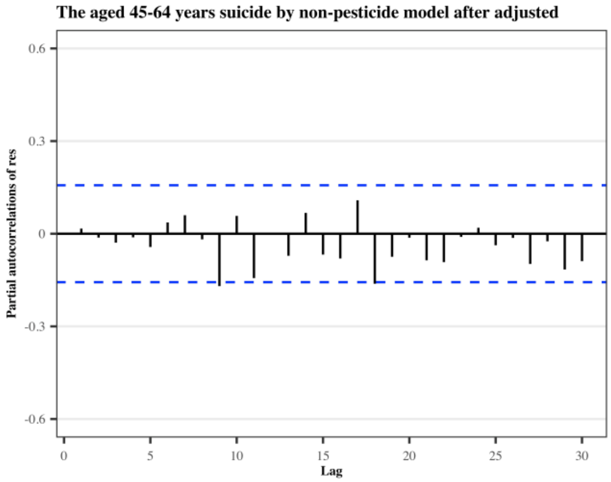
The aged 45-64 years suicide by non-pesticide model after adjusted
Partial autocorrelations of res
Lag
0.6
0.3
0
-0.3
-0.6
0
5
10
15
20
25
30

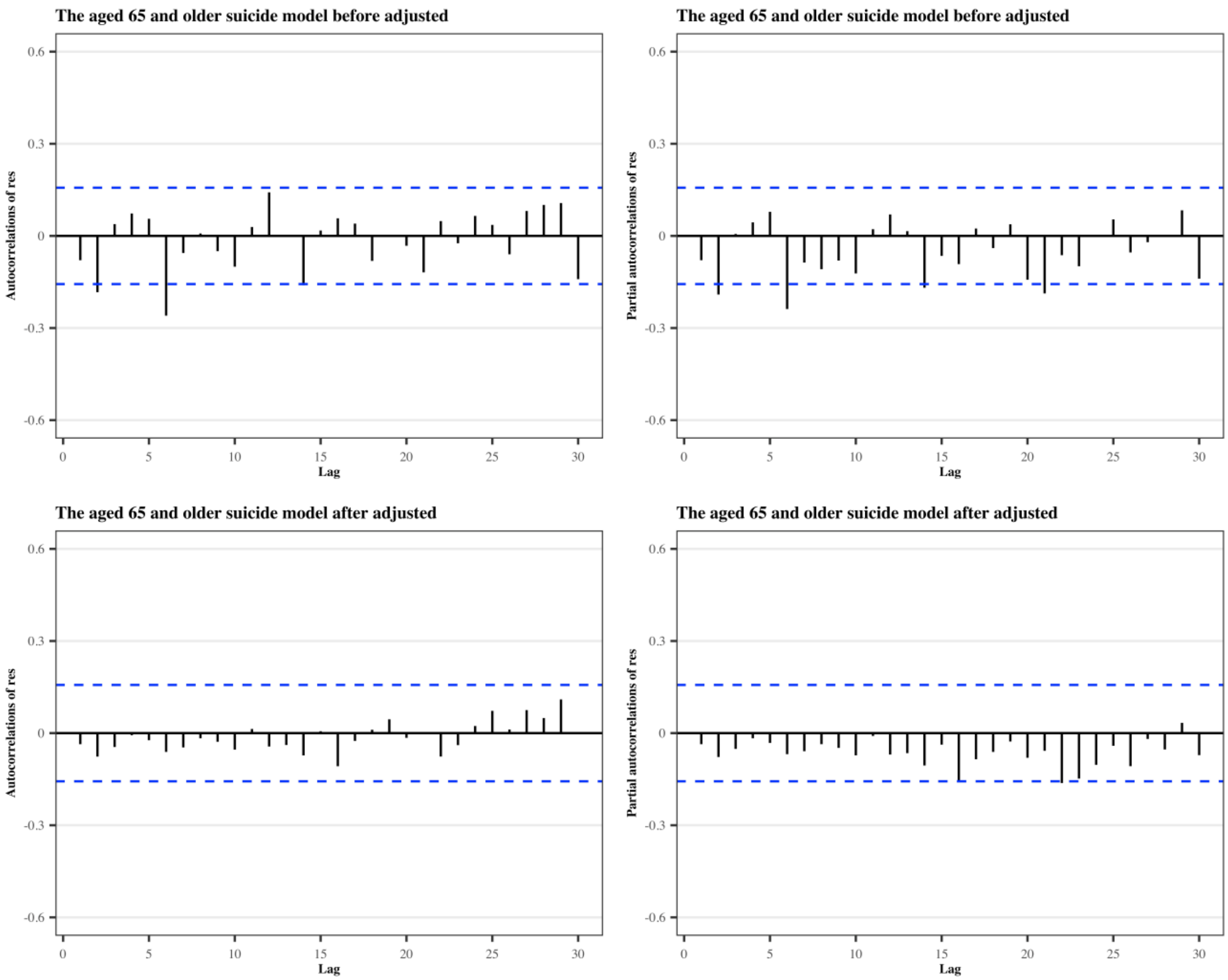

The aged 65 and older suicide model before adjusted
Autocorrelations of res
Partial autocorrelations of res
The aged 65 and older suicide model after adjusted
Autocorrelations of res
Partial autocorrelations of res
0.6
0.3
0
-0.3
-0.6
0
5
10
15
20
25
30
Lag

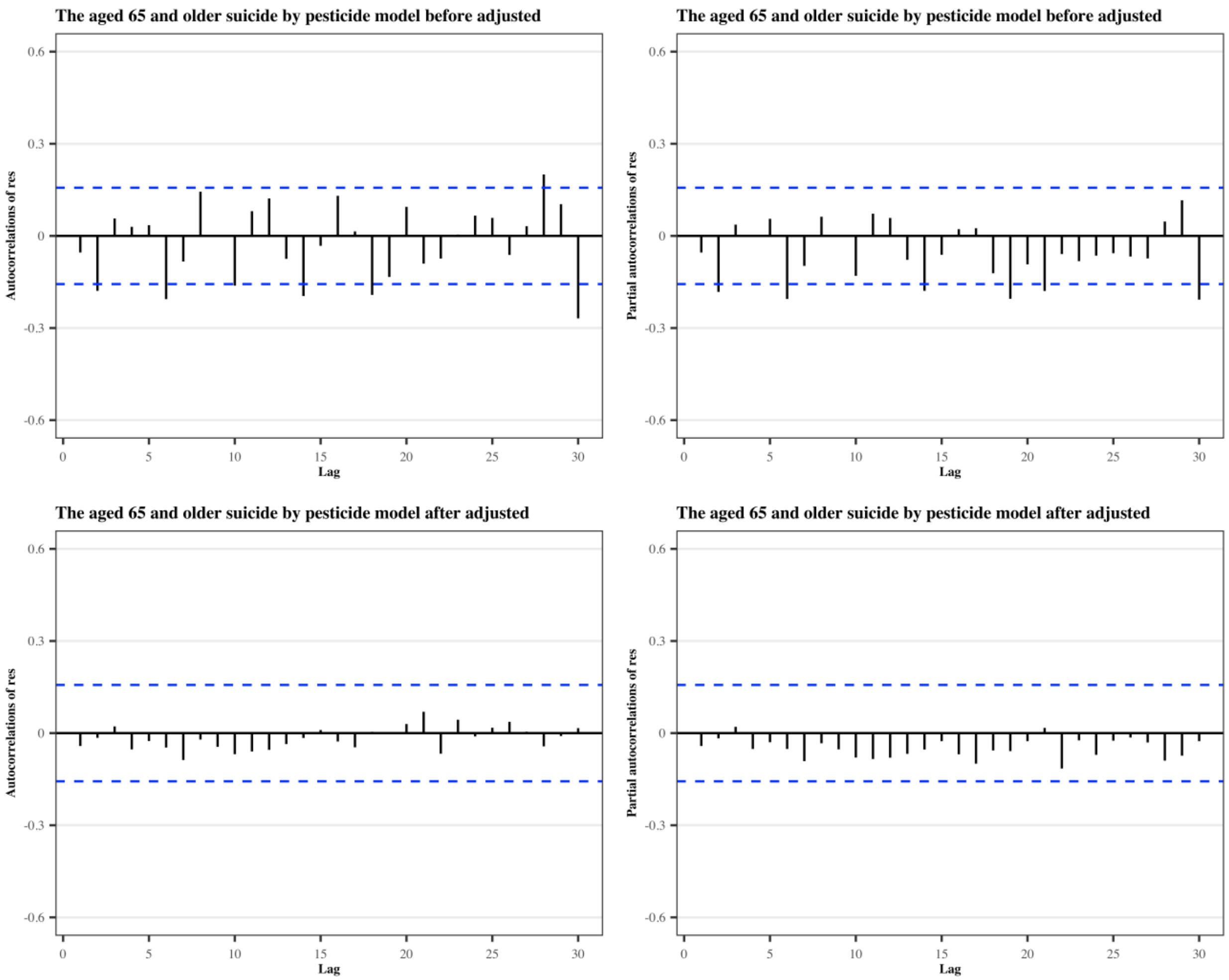
The aged 65 and older suicide by pesticide model before adjusted
Autocorrelations of res
Partial autocorrelations of res
The aged 65 and older suicide by pesticide model after adjusted
0.6
0.3
0
-0.3
-0.6
0
5
10
15
20
25
30
Lag

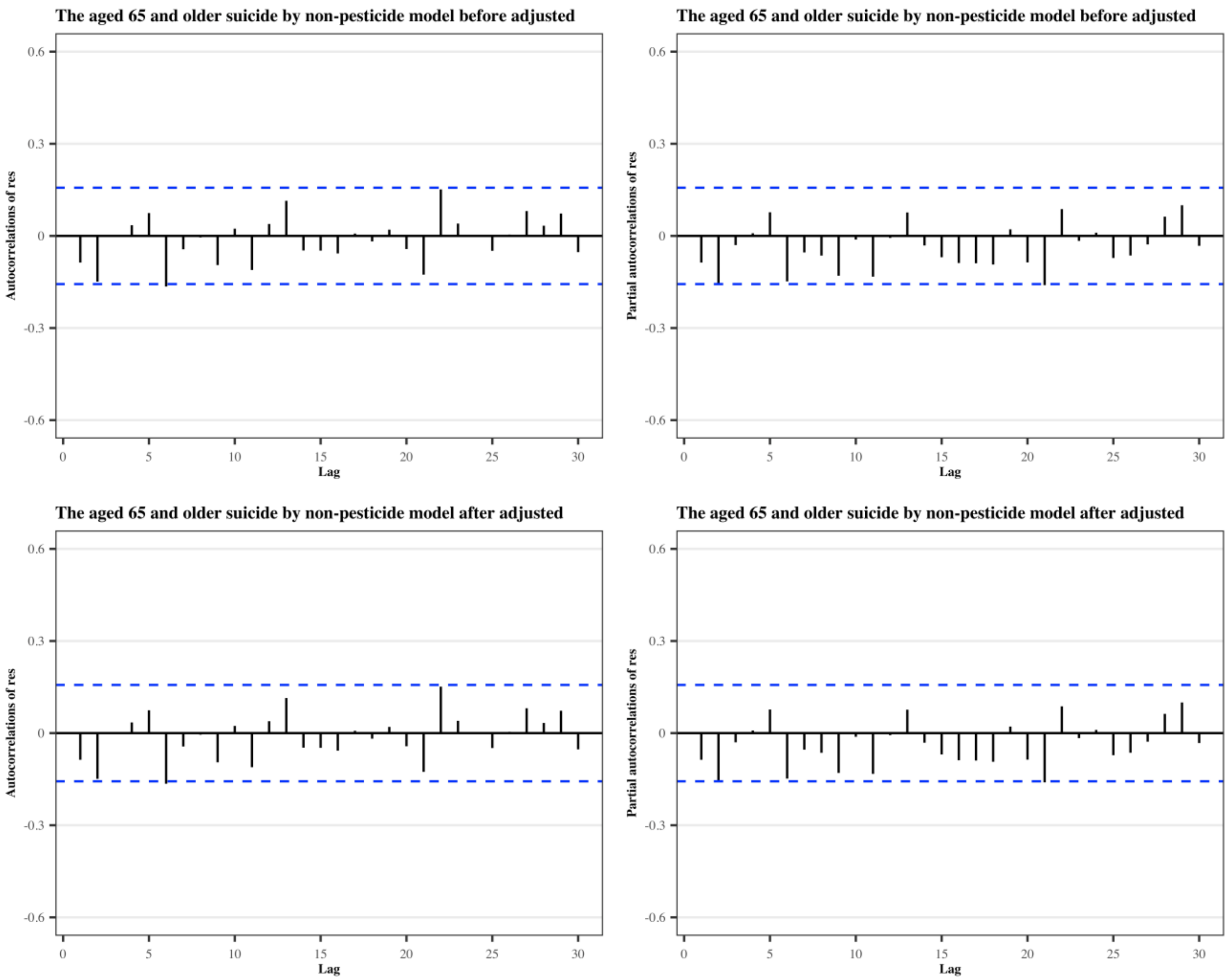
The aged 65 and older suicide by non-pesticide model before adjusted
Autocorrelations of res
Partial autocorrelations of res
The aged 65 and older suicide by non-pesticide model after adjusted
Autocorrelations of res
Partial autocorrelations of res
Lag
0.6
0.3
0
-0.3
-0.6
0
5
10
15
20
25
30
